# Circumvention of Mcl-1-Dependent Drug Resistance by Simultaneous Chk1 and MEK1/2 Inhibition in Human Multiple Myeloma Cells

**DOI:** 10.1371/journal.pone.0089064

**Published:** 2014-03-04

**Authors:** Xin-Yan Pei, Yun Dai, Jessica Felthousen, Shuang Chen, Yukie Takabatake, Liang Zhou, Leena E. Youssefian, Michael W. Sanderson, Wesley W. Bodie, Lora B. Kramer, Robert Z. Orlowski, Steven Grant

**Affiliations:** 1 Division of Hematology/Oncology, Department of Medicine, Virginia Commonwealth University and the Massey Cancer Center, Richmond, Virginia, United States of America; 2 Department of Lymphoma/Myeloma, the University of Texas MD Anderson Cancer Center, Houston, Texas, United States of America; 3 Department of Biochemistry, Virginia Commonwealth University and the Massey Cancer Center and Institute of Molecular Medicine, Richmond, Virginia, United States of America; University of Illinois at Chicago, United States of America

## Abstract

The anti-apoptotic protein Mcl-1 plays a major role in multiple myeloma (MM) cell survival as well as bortezomib- and microenvironmental forms of drug resistance in this disease. Consequently, there is a critical need for strategies capable of targeting Mcl-1-dependent drug resistance in MM. The present results indicate that a regimen combining Chk1 with MEK1/2 inhibitors effectively kills cells displaying multiple forms of drug resistance stemming from Mcl-1 up-regulation in association with direct transcriptional Mcl-1 down-regulation and indirect disabling of Mcl-1 anti-apoptotic function through Bim up-regulation and increased Bim/Mcl-1 binding. These actions release Bak from Mcl-1, accompanied by Bak/Bax activation. Analogous events were observed in both drug-naïve and acquired bortezomib-resistant MM cells displaying increased Mcl-1 but diminished Bim expression, or cells ectopically expressing Mcl-1. Moreover, concomitant Chk1 and MEK1/2 inhibition blocked Mcl-1 up-regulation induced by IL-6/IGF-1 or co-culture with stromal cells, effectively overcoming microenvironment-related drug resistance. Finally, this regimen down-regulated Mcl-1 and robustly killed primary CD138^+^ MM cells, but not normal hematopoietic cells. Together, these findings provide novel evidence that this targeted combination strategy could be effective in the setting of multiple forms of Mcl-1-related drug resistance in MM.

## Introduction

Multiple myeloma (MM) is a clonal accumulative disease of mature plasma cells which, despite recent treatment advances, is generally fatal [1], [2]. As in numerous other malignancies, MM is characterized by dysregulation of apoptotic regulatory proteins of the Bcl-2 family [3], [4]. Among these, the anti-apoptotic protein Mcl-1, encoded by the Mcl-1 (myeloid leukemia cell-1) gene located on chromosome 1q21, has been implicated in the pathogenesis of various malignancies, particularly MM [5], [6]. Mcl-1 promotes proliferation, tumorigenesis, and drug resistance of MM cells [3], [5]. Notably, whereas Mcl-1 represents a factor critical for MM cell survival [4], it has also been shown to confer resistance to the proteasome inhibitor bortezomib, one of the most active agents in current MM therapy [7]–[9]. Of note, Mcl-1 is over-expressed in cells from MM patients, and correlates with relapse and short survival [10]. Moreover, it is widely recognized that the bone marrow microenvironment (BMME) plays an important role in MM cell survival [2], [11], [12]. Furthermore, tumor-microenvironment interactions confer drug resistance to diverse drug classes [13], [14] and may limit the translational potential of promising pre-clinical approaches [11], [15]. Consequently, therapeutic strategies targeting tumor-microenvironment interactions represent an area of intense interest in MM [12], [16]. Significantly, several studies suggest that Mcl-1 also plays an important role in microenvironment-related form of drug resistance in MM [9], [17], [18].

Mcl-1 pro-survival activities have been primarily attributed to interactions with pro-apoptotic Bcl-2 family members such as Bak and Bim [19], [20], although this protein binds to multiple Bcl-2 family members. Mcl-1 expression is regulated at the transcriptional, translational, and post-translational levels [21], and is distinguished by a short half-life (e.g., 30 min to 3 h.) [5], [6]. This has prompted efforts to down-regulate Mcl-1 expression in MM and other Mcl-1-related malignancies e.g., utilizing CDK inhibitors/transcriptional repressors [20], [22] or translational inhibitors (e.g., sorafenib) [23], among others. An alternative strategy involves the use of BH3 mimetics which bind to and inactivate multi-domain anti-apoptotic proteins. While some of these (e.g. ABT-737 or ABT-199) display low avidity for and minimal activity against Mcl-1 [24], [25], others, including pan-BH3 mimetics such as obatoclax, act against this protein [19], [26]. However, the latter agent is no longer being developed clinically. Moreover, questions have arisen regarding the specificity of putative Mcl-1 antagonists [27]. Collectively, these considerations justify the search for alternative strategies capable of circumventing Mcl-1-related drug resistance.

Chk1 is a protein intimately involved in the DNA damage response [28], [29]. Exposure of MM cells to Chk1 inhibitors induces MEK1/2/ERK1/2 activation through a Ras- and Src-dependent mechanism. Moreover, interrupting this event by clinically relevant agents targeting the Src/Ras/MEK/ERK pathway synergistically induces MM cell apoptosis *in vitro* and *in vivo* [28], [30], [31]. Evidence that interruption of the MEK1/2/ERK1/2 pathway down-regulates Mcl-1 expression [32] and/or alters its associations with pro-apoptotic effectors (e.g., Bak and Bim) [33]–[35] raised the possibility the Chk1/MEK1/2 inhibitor strategy might be active in the face of Mcl-1-related forms of drug resistance in MM. However, no information is currently available concerning whether this strategy would be effective in this setting, and if so, by what mechanism(s). Here we report that Chk1/MEK1/2 inhibition induces pronounced apoptosis in bortezomib-resistant MM cells exhibiting Mcl-1 up-regulation, and overcomes drug resistance stemmed from IL-6, IGF-1, or stromal cells. The present findings also suggest two distinct but interrelated mechanisms by which this strategy may target Mcl-1, including transcriptional down-regulation of Mcl-1 and inhibition of its anti-apoptotic function. Collectively, these findings highlight an alternative approach to circumventing Mcl-1-dependent bortezomib- and microenvironment-related drug resistance in MM.

## Materials and Methods

### Cells and reagents

Human MM cell lines U266 and NCI-H929, and human bone marrow stromal cell (BMSC) line HS-5 were purchased from ATCC and maintained as described previously [36]. RPMI8226 cells were from Dr. Alan Lichtenstein (University of California, Los Angeles) [37]. Dexamethasone-sensitive (MM.1S) and -resistant (MM.1R) cell lines were provided by Dr. Steven T. Rosen (Northwestern University, Chicago, IL) [38]. U266/Mcl-1 and RPMI8226/Mcl-1 cells were established by stably transfecting with a construct encoding human full-length Mcl-1 as before [20]. Bortezomib-resistant U266 cells (PS-R) [20] and OPM2 cells (V10R) [39] were generated and maintained as described previously, all experiments were performed using logarithmically growing cells (3–6×10^5^cells/ml).

BM samples were obtained with written informed consent according to the Declaration of Helsinki from nine MM patients undergoing routine diagnostic aspiration with VCU IRB approval. CD138^+^ and CD138^−^ cells were separated using the MACS magnetic separating system according to the manufacturer's instructions (Miltenyi Biotech, Auburn, CA) [30]. Briefly, mononuclear cells were isolated from bone marrow samples by Ficoll-Hypaque (Sigma, St Louis, MO), and then incubated with MACS CD138 microbeads at 4°C for 15 minutes. CD138^+^ cells were then isolated using an MS^+^/LS^+^ column and a magnetic separator. The purity of CD138^+^ cells (>90%) was determined by CD138-PE staining and flow cytometry. Viability (>95%) of both CD138^+^ and CD138^−^ cells was assessed by trypan blue exclusion. Isolated cells were maintained in RPMI 1640 medium containing 10% FCS in 96-well plates. Normal BM CD34^+^ cells were purchased from Lonza (Walkersville, MD). Purity of CD34^+^ cells was >95% and viability >80% when thawed.

The pre-clinical Chk1 inhibitor CEP3891 [29], [36] was provided by Cephalon. The MEK1/2 inhibitor PD184352 (formerly Upstate Biotech, now Millipore) [40], analogous to the first MEK1/2 inhibitor (PD325901) to be used in humans. Dexamethasone and melphalan were purchased from Sigma (St. Louis, MO). Reagents were dissolved in sterile DMSO (final concentration <0.1%). Melphalan was dissolved in HCl-ethanol. Recombinant human IL-6 and IGF-I were purchased from Sigma (St. Louis, MO) and R&D Systems (Minneapolis, MN) respectively, rehydrated in PBS and 10 mM acetic acid (containing 0.1% BSA). All reagents were stored at −80°C.

### Analysis of effects of the microenvironment on MM cell viability

To assess effects of stromal cells on drug activity, a co-culture model of MM cells with human BMSCs (HS-5) was employed [11], [41], [42]. Briefly, MM cells were stably transfected with a construct expressing luciferase (Luc) or GFP (phrGFP Vector, Agilent Technologies) [20]. HS-5 cells were pre-plated for 48 h on multi-well plates or Lab-Tek Chamber Slide System (Nalge Nunc, Naperville, IL), followed by seeding Luc- or GFP-expressing MM cells and co-culturing for an additional 24 h. After drug treatment (48 h), cells were subjected to the following analyses: a) bioluminescent assay using luciferin (RPI, Mount Prospect, IL) by Envision Multilabel Reader (PerkinElmer, Waltham, MA); b) flow cytometry after staining with 7AAD; c) microphotography using an Olympus IX71 Inverted Fluorescence Microscope with CS-DIM imaging software (Olympus, Centervalley, PA) after 7AAD (0.5 µg/ml) staining at 37°C for 20 min; d) assessment of colony-forming ability after 3 weeks by fluorescence microscopy as above (colonies were defined as clusters of >50 GFP^+^ cells); or e) Western blot analysis. In parallel, HS-5-conditional medium was prepared and used as described previously [20].

### Western blot analysis

Whole-cell lysates were extracted using Triton X-100 lysis buffer containing 1% Triton X-100, 50 mM HEPES (pH 7.5), 5 mM EDTA, 50 mM NaCl, 10 mM sodium pyrophosphate, 50 mM sodium fluoride, 1 mM Na_3_VO_4_, 1 mM phenylmethylsulfonyl fluoride, 10 µg/ml aprotinin, and 10 µg/ml leupeptin). Protein samples were harvested as the supernatant following centrifugation at 12,800 *g* for 5 minutes [40]. Alternatively, subcellular fractions were prepared as follows. 4×10^6^ cells were washed in PBS and lysed by incubating in digitonin lysis buffer (75 mM NaCl, 8 mM Na_2_HPO_4_, 1 mM NaH_2_PO_4_, 1 mM EDTA, and 350 µg/ml digitonin) for 30 seconds. After centrifugation at 12,000 *g* for 1 minute, the supernatant (S-100 cytosolic fraction) was collected in an equal volume of 2×sample buffer. The pellets (organelle/membrane fractions) were then washed once in cold PBS and lysed in 1× sample buffer.

The amount of total protein was quantified using Coomassie protein assay reagent (Pierce, Rockford, IL). 20 µg of protein were separated on precast SDS-PAGE gels (Invitrogen, CA) and electrotransferred onto nitrocellulose membranes. Blots were reprobed with antibodies against β-actin (Sigma) or α-tubulin (Oncogene, La Jolla, CA) to ensure equal loading and transfer of proteins. Blots were probed with primary antibodies including: anti-Mcl-1, anti–caspase-3, and anti–cytochrome c (BD Biosciences, San Jose, CA); anti-Bim and anti–smac/DIABLO (Millipore, Billerica, MA); anti-PARP (Biomol, Plymouth Meeting, PA); anti-cleaved caspase-3 and anti-phospho-p44/42 (Thr202/Tyr204) MAPK (Cell Signaling, Beverly, MA); anti-Bax (Santa Cruz Biotechnology, Santa Cruz, CA).

### Immunoprecipitation (IP)

Interactions between Mcl-1 and Bim or Bak were evaluated by co-IP analysis. CHAPS buffer (150 mmol/L NaCl, 10 mmol/L HEPES [N-2-hydroxyethylpiperazine-N′-2-ethanesulfonic acid] pH 7.4, protease inhibitors, and 1% CHAPS) was employed to avoid artifactual associations [43]. Cells were lysed in CHAPS buffer and 200 µg of protein per condition were immunoprecipitated with 1 µg anti-Mcl-1 (Santa Cruz Biotechnology or BD Biosciences), anti-Bak, or anti-Bim (Santa Cruz Biotechnology), followed by Dynabeads (Dynal, Oslo, Norway). IP samples were then subjected to Western blot analysis using anti-Bim (Millipore), anti-Mcl-1, or anti-Bak (Santa Cruz Biotechnology) as primary antibodies, respectively.

To monitor Bak and Bax conformational change, anti-Bax (6A7, Sigma) or anti-Bak (Ab-1, Millipore) antibodies, which only recognize Bax or Bak that have undergone conformational change, were used for IP, followed by Western blot analysis using anti-Bax and anti-Bak as primary antibodies.

### Quantitative real time-PCR (qRT-PCR)

Quantitative PCR (qPCR) analysis using TaqMan gene expression assay (assay ID, Hs03043899_m1) and 7900HT real-time PCR system (Applied Biosystems, Foster City, CA) were employed to quantify human Mcl-1 mRNA [20]. Human GAPDH (Pre-Developed TaqMan Assay Reagents Control Kit) was used as reference for quantitation. Data was analyzed using SDS 2.3 software.

### Flow cytometry

Apoptosis was monitored by annexin V-FITC staining and flow cytometry. Primary MM cell viability was determined by trypan blue exclusion. MM cell death was also monitored by 7-AAD staining (0.5 µg/mL at 37°C for 30 min). Cell death of MM cells co-cultured with HS-5 stromal cells was determined by monitoring the percentage of 7AAD^+^ cells in the GFP^+^ gated population (i.e., myeloma cells labeled with GFP) by flow cytometry.

### Clonogenic assays

Colony-forming ability was evaluated using a previously described soft agar cloning assay [30]. In brief, U266 cells, with or without HS-5 cells, were treated with 400 nM CEP3891±7.5 µM PD184352 for an additional 48 h, after which, cells were washed free of drug and plated in soft agar for 21 days. Colonies, consisting of groups of >50 myeloma cells, were then scored for each condition. In this system, the morphology of myeloma cell colonies with or without HS-5 cells was identical, and clearly distinguishable from HS-5 colonies. In addition, colonies were stained with 0.1% crystal violet for 3 hrs and images captured by digital camera (Model: Power shot A640). To confirm myeloma cell colony-forming ability of cells co-cultured with HS-5 cells, the colony-forming ability of GFP^+^ U266 cells was monitored by fluorescence microscopy; colonies were defined as clusters of >50 green fluorescent protein–positive (GFP^+^) cells. Within the same field, bright field images were captured for all colonies, including HS-5 cells.

### Statistical analysis

Values represent the means ± SD for at least 3 separate experiments performed in triplicate. Significance of differences between experimental variables was determined using the Student's t test. Median dose effect analysis [44] of apoptosis induction by PD184352 and CEP3891 administered over a range of concentrations at a fixed ratio was performed to assess synergism using the software program Calcusyn (Biosoft, Ferguson, MO) according to the manufacturer's instructions. CI values less than 1.0 indicate synergism.

## Results

### Simultaneous inhibition of Chk1 and MEK1/2 down-regulates Mcl-1 and effectively induces apoptosis in MM cells

**Figure 1:**
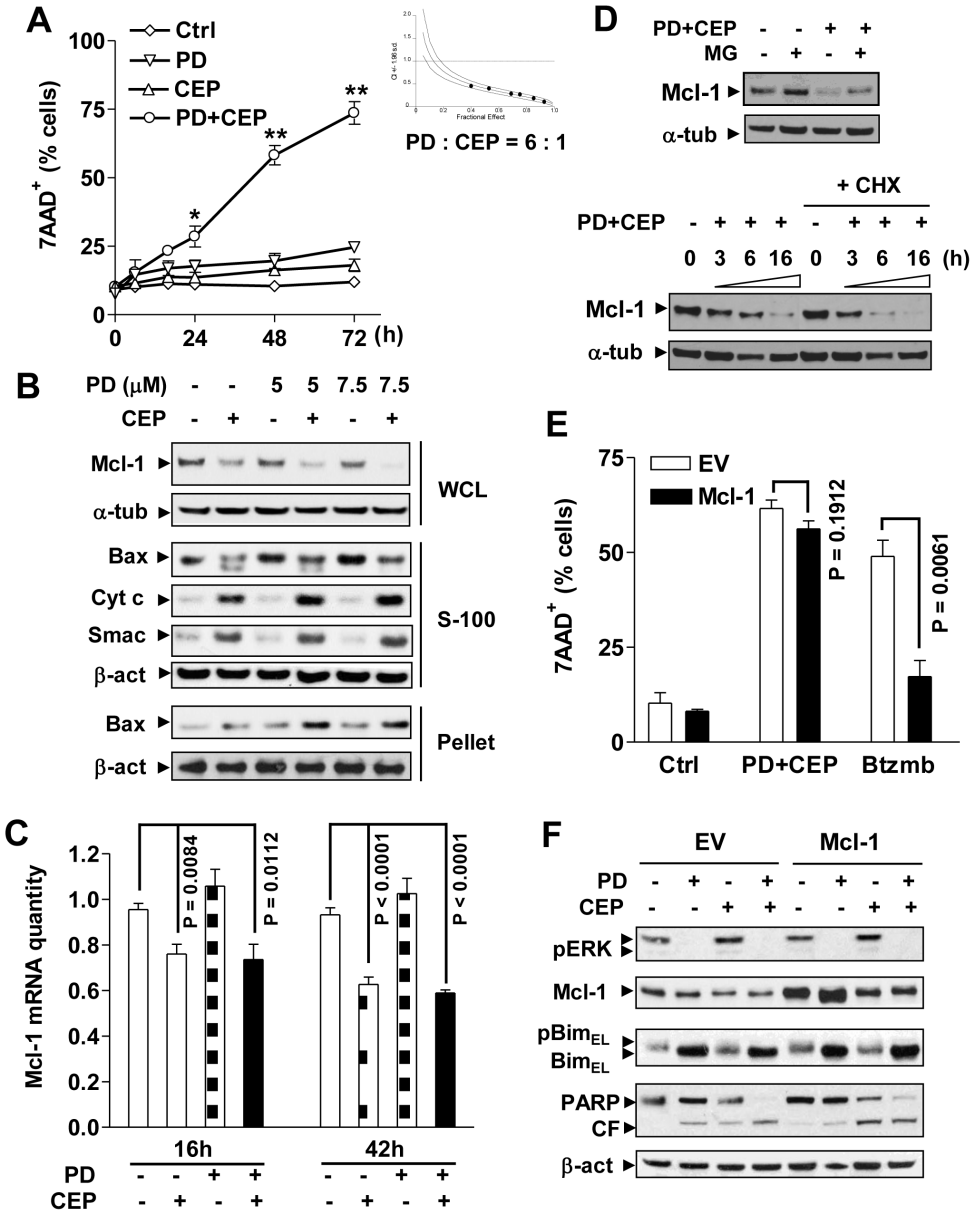
Combined treatment with CEP3891/PD184352 down-regulates Mcl-1 and induces apoptosis in MM cells including those ectopically over-expressing Mcl-1. (**A**) U266 cells were co-exposed to 400 nM CEP3891±7.5 µM PD184352 for indicated interval, after which cell death was monitored by flow cytometry. Values represent the means and SD for three separate experiments performed in triplicate (* P<0.01 or ** P<0.001). U266 cells were treated (48 h) with a range of concentrations of CEP3891±PD184352 at a fixed ratio (6∶1), after which median dose effect analysis was used to characterize the nature of the interaction (inset). (**B**) U266 cells were treated with 400 nM CEP3891±PD184352 at the indicated concentrations for 16 h, after which subcellular fractions were prepared as discribed in meterial and method. Western blot analysis was performed using the indicated primary antibodies. WCL, whole cell lysate; S-100, cytosol; pellet, mitochondria-enriched fraction; Cyto c = cytochrome c. For these and all subsequent Western blot analyses, each lane was loaded with 20 µg of protein; blots were stripped and re-probed with α-tubulin (α-tub) or β-actin (β-act) antibodies to ensure equal loading and transfer; two additional studies yielded equivalent results. (**C**) U266 cells were treated with 400 nM CEP3891±7.5 µM PD184352 for 16 and 42 h, after which real-time qRT-PCR was performed to quantify Mcl-1 mRNA. Values represent the means and SD for three separate experiments. (**D**) U266 cells were incubated with 400 nM CEP3891±7.5 µM PD184352 in the presence or absence of 300 nM MG-132 (16 h, upper) or 1 µM CHX (3, 6, and 16 h, lower), after which protein levels of Mcl-1 were assessed by Western blot analysis. (**E**) U266 cells were stably transfected with a construct encoding human full-length Mcl-1 or empty vector (EV). Following treatment with 500 nM CEP3891±7.5 µM PD184352 for 48 h, the percentage of dead cells was determined by flow cytometry. In parallel, empty-vector and Mcl-1 over-expressing U266 were treated with 5 nM bortezomib as a control. Values represent the means and SD for three separate experiments performed in triplicate. (**F**) Alternatively, cells were subjected to Western blot analysis using the indicated primary antibodies. CF, cleavage fragment.

Because UCN-01 displays off-target effects towards multiple proteins including PKC, CDKs, and PDK1 [45], a newer generation of more specific Chk1 inhibitors (e.g., CEP3891) have recently been developed [29]. U266 cells exposed to 400 nM CEP3891 (48 h) experienced minimal toxicity, while combined treatment with a sub-toxic concentration of the MEK1/2 inhibitor PD184352 (7.5 µM) synergistically increased cell death (**Fig. 1A** and **Fig. S1A**), with combination index values less than 1 over a range of concentrations by Median Dose Effect Analysis (inset). Dose response analysis yielded consistent results (**Fig. S1B**, **C**). Similar interactions also occurred in multiple other MM cell lines, including H929, MM1.S, MM1.R, and 8226 (**Fig. S1D–F**). Exposure of U226 cells to CEP3891 down-regulated Mcl-1, an event enhanced when combined with PD184352, accompanied by increased Bax mitochondrial translocation, cytosolic release of cytochrome C and Smac (**Fig. 1B**), and cleavage of caspase-3 and PARP (**Fig. S1G**). Similarly, Mcl-1 down-regulation and increased caspase 3 cleavage following combined treatment were observed in several other MM lines e.g., 8266, H929, MM1.S, and OPM2 (**Fig. S2A**). Interestingly, qRT-PCR revealed that while PD184352 modestly increased mRNA levels of Mcl-1, CEP3891 alone or in combination partially but significantly reduced Mcl-1 mRNA levels at 6, 16, and 42 h in U266 cells, compared to untreated control (**Fig. 1C** and **Fig. S2B**). However, inhibition of protein translation by CHX or proteasomal degradation by MG-132 had a little effect on Mcl-1 down-regulation by CEP3891 in combination with PD184352 (**Fig. 1D** and **Fig. S2C**). On the other hand, exposure to PD184352 markedly up-regulated Bim (**Fig. S1G**), as described earlier [40]. Together, these results raise the possibility that whereas CEP3891 down-regulates Mcl-1 and PD187352 up-regulates Bim, the anti-MM activity of this combination regimen may involve cooperative effects of these two events.

### Ectopic overexpression of Mcl-1 fails to protect MM cells from the MEK/Chk1 inhibition strategy

To assess effects of simultaneous MEK/Chk1 inhibition on MM cells overexpressing Mcl-1, U266 cells ectopically expressing Mcl-1 (U266/Mcl-1) were employed. In contrast to pronounced resistance of U266/Mcl-1 cells to bortezomib (P<0.01 vs empty vector control U266/EV), the CEP3891/PD184352 regimen induced equivalent apoptosis in both cell lines (P>0.05, **Fig. 1E**). Interestingly, as shown in **Fig. 1F**, CEP3891 alone or in combination clearly down-regulated Mcl-1, while PD184352 up-regulated Bim presumably via ERK1/2 inactivation, together markedly increasing PARP cleavage in both empty vector control (U266/EV) and U266/Mcl-1 cells. Similar results were obtained in 8226 cells ectopically overexpressing Mcl-1 (**Fig. S2D**, **E**). These findings argue that Mcl-1 over-expression, which confers marked resistance to bortezomib, does not confer cross-resistance to the MEK/Chk1 inhibitor regimen.

### Increased binding of Bim to Mcl-1 is associated with release of Bak from Mcl-1 following combined Chk1/MEK1/2 inhibitor treatment

**Figure 2:**
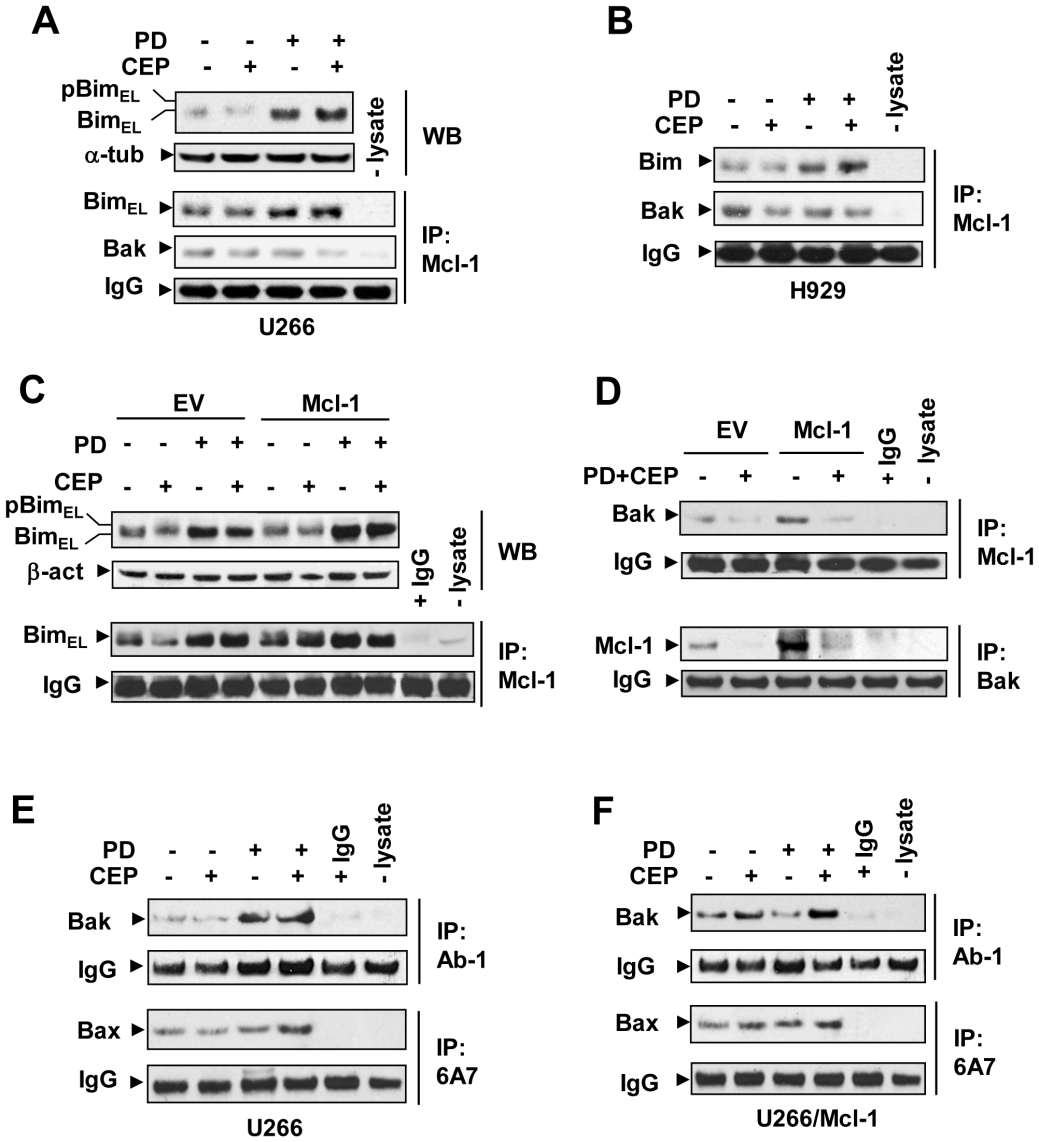
The PD184352/CEP3891 regimen increases Bim/Mcl-1 binding, releases Bak from Mcl-1, and triggers Bak/Bax activation. (**A**) and (**B**) U266 (**A**) and H929 (**B**) cells were exposed to 500 nM CEP3891±PD184352 (7.5 µM for U266; 2.5 µM for H929) for 42 h; (**C**) and (**D**) U266 cells over-expressing Mcl-1 and their EV controls were treated as described for U266 in panel 2A. After treatment, cells were lysed in 1% CHAPS buffer and immunoprecipitated (IP) using anti-Mcl-1 (**C**) or anti-Bak (**D**) antibodies, followed by Western blot (WB) analysis using anti-Bim, anti-Bak, or anti-Mcl-1 antibodies as indicated. WCL were loaded to monitor Bim levels. (**E**) and (**F**) Alternatively, following 24 h treatment, IP was performed to monitor conformational change of Bax and Bak using anti-Bax 6A7 or anti-Bak Ab-1 (for IP), and anti-Bax or anti-Bak (for WB) in parental U266 cells (**E**) and their counterparts ectopically expressing Mcl-1 (**F**). For all IP assays, IPs without cell lysate (-lysate) and/or with IgG (instead of primary antibodies) were carried out as controls; 200 µg protein per condition were employed for IP; IgG levels are shown to ensure equal loading of IP antibodies.

In view of evidence that in addition to the relative protein levels of pro-and anti-apoptotic Bcl-2 family proteins, interactions between these agents may also be involved in determination of cell fate [46], associations between Mcl-1 and Bim or Bak were then examined. PD184352+/−CEP3891 increased the amount of Bim co-immunoprecipitating with Mcl-1, presumably due to Bim up-regulation, accompanied by dissociation between Mcl-1 and Bak (**Fig. 2A, B**). Similar phenomena were also observed in Mcl-1-overexpressing cells (**Fig. 2C, D**). Moreover, combined treatment induced activation of both Bak and Bax in parental U266 cells (**Fig. 2E**) as well as in their counterparts ectopically expressing Mcl-1 (**Fig. 2F**). Release of Bak from Mcl-1 also led to its activation in 8226 cells ectopically over-expressing Mcl-1 (**Fig. S2F**). To test the possibility that Mcl-1 may also be disabled through Noxa up-regulation, as reported in the case of bortezomib [47], Western blot analysis was performed to monitor Noxa expression in various MM cell lines. However, in contrast to findings involving bortezomib, these studies revealed no clear induction of Noxa following exposure to PD184352 alone or in combination with CEP3891 (**Fig. S3A**). These findings support the notion that the MEK/Chk1 inhibitor regimen up-regulates Bim and increases binding of Bim to Mcl-1, leading to Bak release from Mcl-1, followed by Bak and Bax activation. Collectively, these findings provide another mechanism, in addition to Mcl-1 down-regulation, that may contribute to circumvention of Mcl-1-dependent drug resistance.

### Bortezomib-resistant MM cells displaying increased Mcl-1 expression do not display cross-resistance to the MEK/Chk1 inhibitor regimen

**Figure 3:**
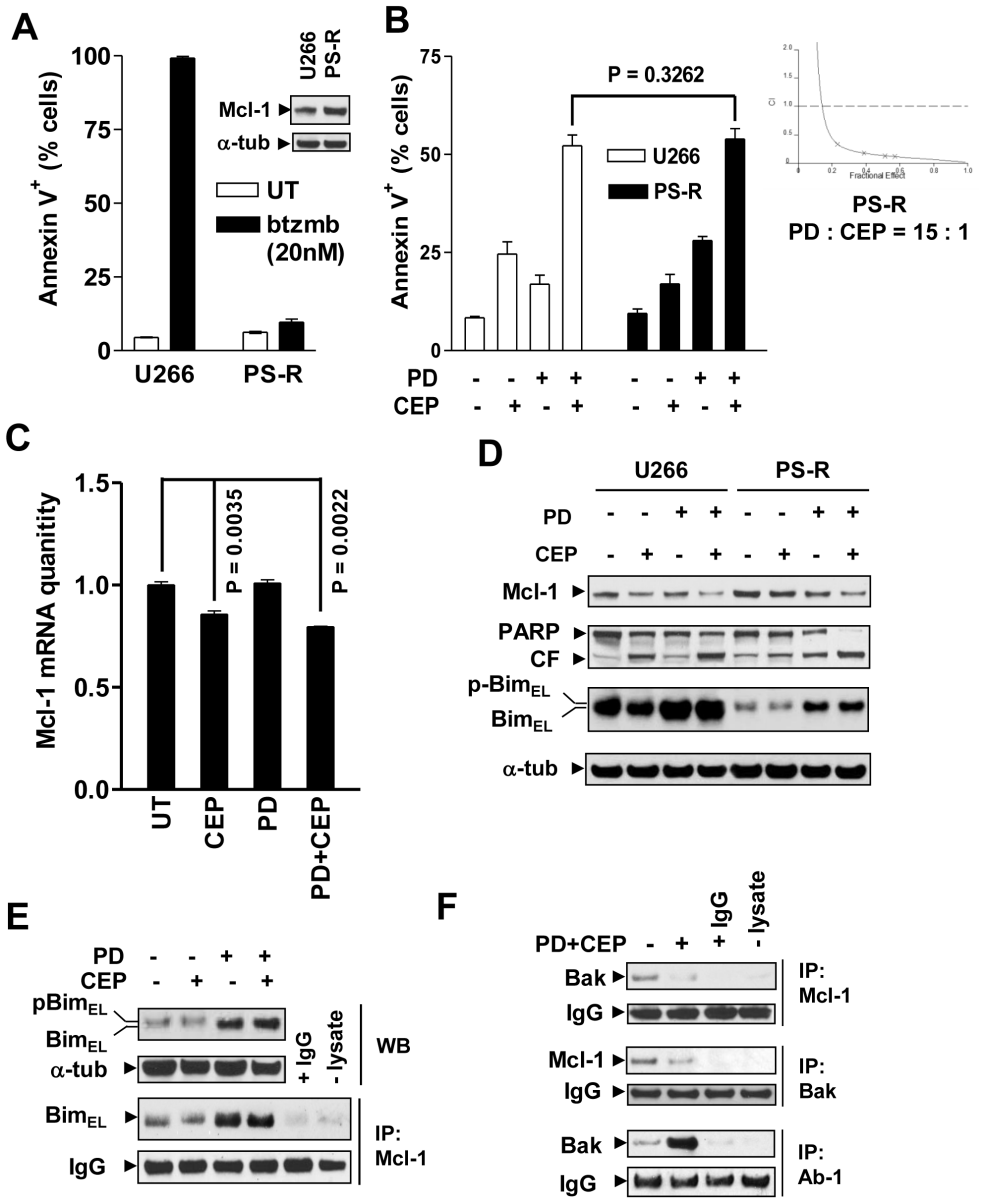
Bortezomib-resistant MM cells exhibiting Mcl-1 up-regulation and Bim down-regulation does not display cross-resistance to PD184352/CEP3891. (**A**) Parental U266 cells and their bortezomib-resistant counterparts (PS-R) were exposed to 20 nM bortezomib (btzmb) for 24 h, after which the percentage of apoptotic cells were determined by Annexin V staining and flow cytometry. Western blot analysis was performed to monitor Mcl-1 protein (inset). (**B**) U266 and PS-R cells were treated with 500 nM CEP3891±7.5 µM PD184352 for 48 h, after which the extent of apoptosis (Annexin V^+^ cells) was determined by flow cytometry. PS-R cells were exposed to a range of concentrations of CEP3891±PD184352 at a fixed ratio (1∶15) for 48 h, after which median dose effect analysis was used to characterize the nature of the interactions using cell death (7AAD^+^) as an endpoint (inset). (**C**) and (**D**) Alternatively, following treatment with 500 nM CEP3891±7.5 µM PD184352, real-time qRT-PCR and Western blot analysis were performed to monitor Mcl-1 mRNA levels (16 h, **C**) and expression of the indicated proteins (40 h, **D**). (**E**) and (**F**) In parallel, following 42 h-exposure to 500 nM CEP3891±7.5 µM PD184352, PS-R cells were subjected to immunoprecipitation (IP) followed by Western blotting to assess interactions between Mcl-1/Bim (**E**) and Mcl-1/Bak (**F**, upper), or Bak conformational change (**F**, lower). WCL was loaded to monitor protein levels. For analyses of flow cytometry and real-time qRT-PCR, values represent the means and SD for three separate experiments.

Parallel studies were performed in bortezomib-resistant U266 cells (PS-R) generated by continuously culturing in progressively increasing bortezomib concentrations. These cells displayed pronounced resistance to bortezomib (**Fig. 3A**) in association with up-regulated Mcl-1 protein (inset) and mRNA levels compared to parental U266 cells (**Fig. S3B**). Notably, these cells were fully sensitive to combined treatment with CEP3891/PD184352 (**Fig. 3B**, P>0.05 vs U266 cells). Moreover, the combination was highly synergistic (CI<0.5) in bortezomib-resistant cells over a range of drug concentrations (inset). Furthermore, exposure to CEP3891 alone or in combination with PD184352 also clearly reduced Mcl-1 mRNA (16 h, **Fig. 3C**; 6 h, **Fig. S3C**, P<0.01 vs untreated control) and protein levels (**Fig. 3D**) in PS-R cells. Moreover, PS-R cells displayed sharply reduced basal Bim levels compared to their parental counterparts. Of note, PD184352 alone or in combination with CEP3891 restored Bim expression. In association with these actions, the combination clearly increased PARP cleavage in both drug-naïve and bortezomib-resistant cell lines (**Fig. 3D**). Lastly, as observed in U266 cells (**Fig. 2A**), PD184352±CEP3891 also increased Bim binding to Mcl-1 (**Fig. 3E**) and released Bak from Mcl-1, leading to Bak activation (**Fig. 3F**), accompanied by Bax mitochondrial translocation, and cytosolic release of cytochrome c and Smac in PS-R cells (**Fig. S3D**). Analogous results were observed in another bortezomib-resistant cell line, OPM2/V10R [39] (**Fig. S4A–D**). Together, these findings argue that the CEP3891/PD184352 regimen transcriptionally down-regulates and functionally disables Mcl-1, and raise the possibility that these events may contribute to the activity of this strategy in bortezomib-resistant MM cells.

### Chk1/MEK1/2 inhibition prevents Mcl-1 up-regulation and circumvents drug resistance induced by growth factors

**Figure 4:**
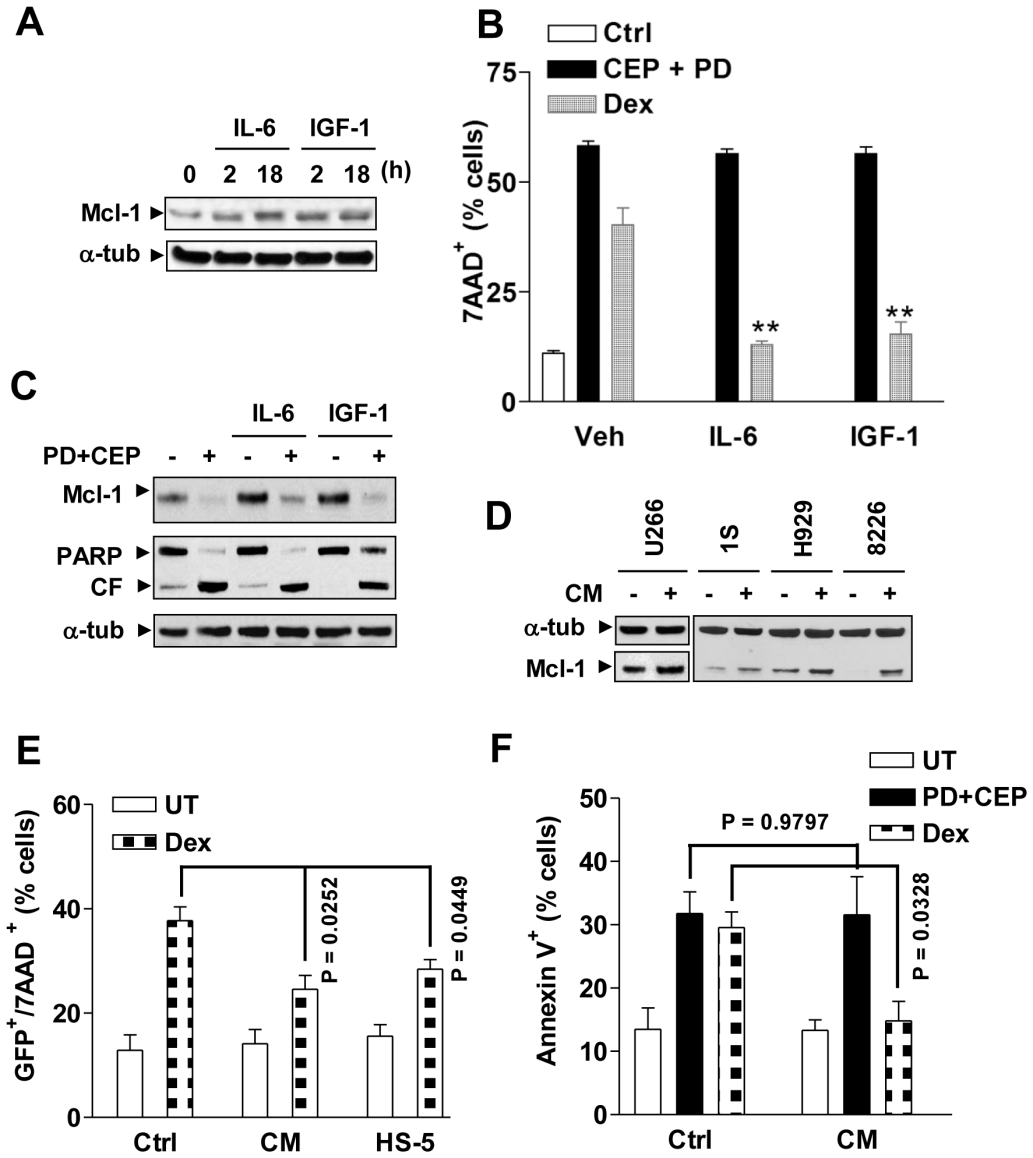
PD184352/CEP3891 attenuates Mcl-1 up-regulation and drug-resistance induced by growth factors. (**A**) U266 cells were cultured in serum-free medium for 6 h, followed by addition of IL-6 (100 ng/ml) or IGF-1 (400 ng/ml) for 2 h and 18 h, after which cells were lysed and subjected to Western blot analysis to assess expression of Mcl-1. (**B**) U266 cells were treated with 400 nM CEP3891+7.5 µM PD184352 or 50 µM dexamathasone (Dex) for 40 h in either the presence or absence of IL-6 or IGF-1. After treatment, the percentage of dead cells was evaluated by flow cytometry (** P<0.01 vs. without IL-6 or IGF-1). (**C**) After pre-incubation with either IL-6 or IGF-1 for 1 h, U266 cells were exposed to 400 nM CEP3891+5 µM PD184352 for 4 h, after which Western blot analysis was performed to monitor Mcl-1 expression and PARP cleavage. (**D**) MM.1S, H929, RPMI8226, and U266 cells were serum-starved for 6 h, and then cultured for an additional 24 h in either fresh 10% FBS medium as a control or conditioned medium (CM) derived from HS-5 cell cultures, after which Western blot analysis cells was performed to assess Mcl-1 expression. (**E**) U266 cells stably expressing GFP were pre-cultured with HS-5 cells or in the presence of HS-5 CM for 48 h, followed by treatment with 50 µM dexamathasone for an additional 40 h. After treatment, the percentage of dead (7AAD^+^) cells in the GFP^+^ population was determined by flow cytometry. (**F**) H929 cells were exposed to 6 µM dexamathasone or 400 nM CEP3891+2.5 µM PD184352 for 38 h in the presence of HS-5 CM, after which the percentage of apoptotic (Annexin V^+^) cells was determined by flow cytometry. For panels 4E and F, Ctrl = 10% FBS medium. For flow cytometry, values represent the means ± SD for three separate experiments.

A link exists between growth factors and Mcl-1 expression in microenvironment-mediated drug resistance to chemotherapeutic agents in MM cells [15], [17], [48]. Consequently, the effects of the MEK/Chk1 inhibitor regimen were examined in MM cells in the presence of growth factors or stromal cell-conditioned medium. Addition of IL-6 or IGF-1 to culture medium induced discernible Mcl-1 up-regulation in MM cells (**Fig. 4A** and **Fig. S4E**) and significantly protected cells from dexamethasone lethality (**Fig. 4B**). In contrast, these growth factors conferred no protection against combined treatment with CEP3891/PD184352 (**Fig. 4B**). Moreover, CEP3891/PD184352 blocked IL-6- and IGF-1-induced Mcl-1 up-regulation, and induced increases in PARP cleavage in either the presence or absence of these growth factors (**Fig. 4C** and **Fig. S4E**). Moreover, conditioned medium derived from human BM stromal HS-5 cells also clearly up-regulated Mcl-1 (**Fig. 4D**), and significantly blocked dexamethasone-induced cell death (**Fig. 4E, F**) as reported earlier [20], [49]. However, conditioned medium was unable to diminish CEP3891/PD184352 lethality in H929 (**Fig. 4F**), U266 (**Fig. S4F**), or 8226 cells (**Fig. S5C**, P>0.05 in each case).

### Stromal cells fail to prevent Mcl-1 down-regulation and MM cell death induced by MEK/Chk1 inhibition

**Figure 5:**
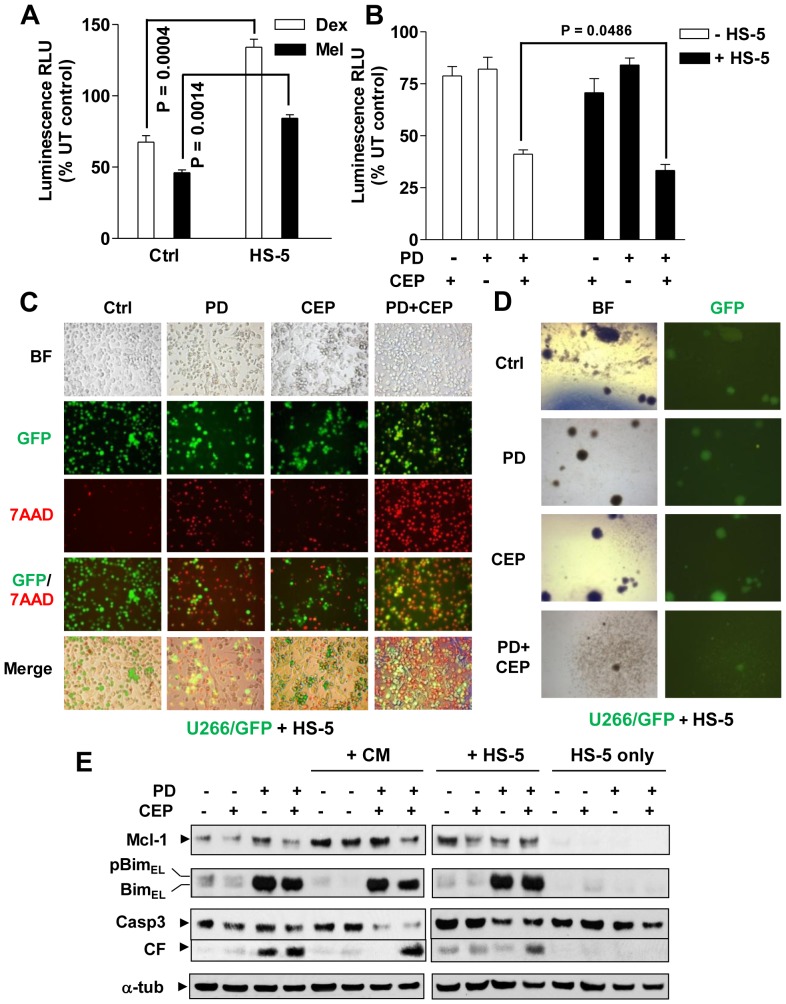
BMSCs fail to protect MM cells from PD184352/CEP3891 lethality. **(A) and (B)** U226 cells stably expressing luciferase were co-cultured for 24 h with HS-5 cells (pre-cultured for 48 h), and then treated with either 50 µM dexamethasone (Dex) or 30 µM melphalan (Mel, **A**) or 400 nM CEP3891±7.5 µM PD184352 (**B**) for an additional 48 h. Bioluminescence intensity, which is proportional to the number of living cells, was monitored to assess cell viability. Values represent the means and SD for three separate experiments performed in triplicate. UT = untreated; RLU = relative light unit. (**C**) GFP-expressing U266 cells were co-cultured for 48 h with HS-5 cells (pre-cultured for 48 h) on the 4-well chamber slides, after which cells were treated with 400 nM CEP3891±7.5 µM PD184352 for an additional 40 h. Cells were then stained with 7AAD and images captured by an inverted fluorescence microscope (Olympus 1X71, 20× objective) with the filters suitable for 7AAD (red) or GFP (green). In parallel, bright field (BF) images were also captured for the same areas. (**D**) After treatment as described in panel 5B, GFP-expressing U266 cells were washed free of drugs and then plated with HS-5 cells on soft agar. After incubation for 21 days, the colony-forming ability of GFP^+^ U266 cells was assessed under fluorescence microscopy (Olympus 1X71, 4× objective); colonies were defined as clusters of >50 GFP^+^ cells. Bright field images were captured for comparison. The microscopic images are representative of three separate experiments. (**E**) H929 cells were treated with 300 nM CEP3891±2.5 µM PD184352 for 48 h under the conditions as follows: a) 10% FBS medium as control (lanes 1–4); b) HS-5-derived conditional medium (CM, lanes 5–8); and c) co-culture with HS-5 (lanes 9–12). In parallel, HS-5 cells alone (lanes 13–16) were treated for comparison. After drug treatment, Western blot analysis was conducted to monitor the expression of Mcl-1 and Bim, as well as caspase 3 cleavage.

To assess effects of interactions with stromal cells on MM cell responses to the MEK/Chk1 inhibitor regimen, MM cells stably expressing luciferase in co-culture with HS-5 cells were used to monitor MM cell viability. Co-culture with HS-5 cells significantly rescued U266 cells (luc^+^) from lethality of dexamethasone or melphalan (**Fig. 5A**), as reported earlier [42]. In sharp contrast, MM cells co-cultured with HS-5 did were, if anything (P<0.05), more sensitive to combined treatment with CEP3891/PD184352 (**Fig. 5B**), reflected by diminished bioluminescent signals proportional to the reduced number of viable cells [11]. Moreover, following CEP3891/PD184352 exposure, fluorescence microscopy revealed a marked increase in 7-AAD uptake (red) by GFP-expressing U266 cells (green) in the presence of HS-5 cells (**Fig. 5C**). This finding was further validated quantitatively by flow cytometry (**Fig. S4F**). Similar data were obtained in luciferase-expressing H929 cells (**Fig. S5A, B**) or GFP-labeled 8226 cells (**Fig. S5C, D**). Importantly, analogous phenomena were also observed in luciferase-expressing bortezomib-resistant PS-R cells (**Fig. S5E**). Finally, co-treatment with CEP3891/PD184352 markedly suppressed colony formation of U266 cells in either the presence or absence of HS-5 cells (**Fig. 5D** and **Fig. S6A–C**).

Expression of Mcl-1 and Bim were then examined in MM cells treated with CEP3891/PD184352 in the presence of HS-5-conditioned medium or HS-5 cells. Notably, CEP3891/PD184352 largely blocked Mcl-1 up-regulation induced by both HS-5 cells and conditioned medium, while up-regulating Bim expression, accompanied by increased caspase-3 cleavage (**Fig. 5E** **and S6D**). Collectively, these findings suggest that MM bone marrow microenvironmental factors are ineffective in protecting MM cells from the MEK/Chk1 inhibitor regimen.

### MEK/Chk1 inhibition down-regulates Mcl-1 and induces cell death in primary MM samples

**Figure 6:**
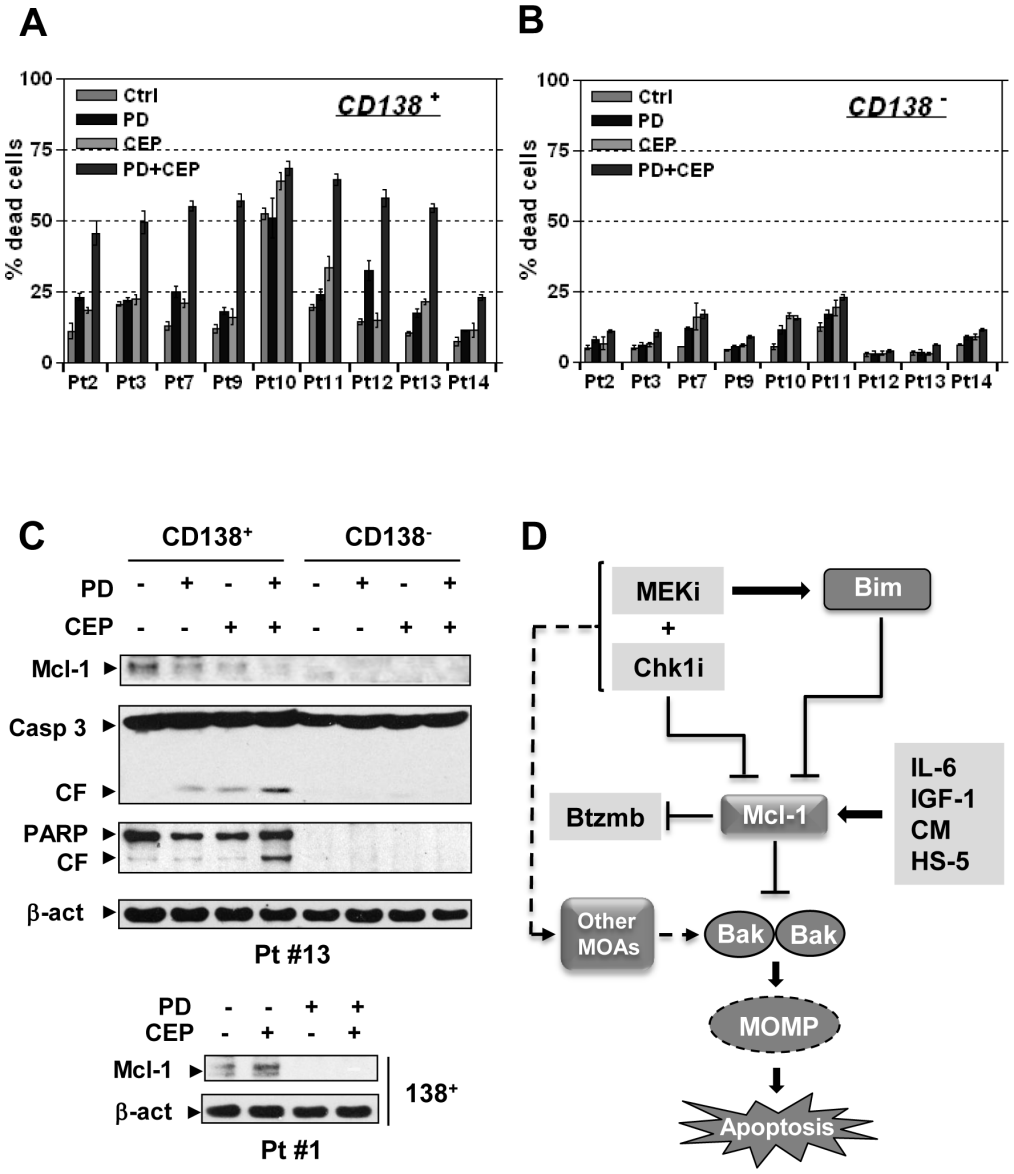
The PD184352/CEP3891 regimen down-regulates Mcl-1 and induces cell death in primary CD138^+^ MM cells. (**A**) and (**B**) Primary CD138^+^ MM cells (**A**) and their normal CD138^−^ counterparts (**B**) were isolated from bone marrow samples obtained from nine patients with MM, and exposed to 500 nM CEP3891±5 µM PD184352 for 24 h. After treatment, cell death was examined by trypan blue exclusion. (**C**) Alternatively, Western blot analysis was performed to monitor expression of Mcl-1 as well as cleavage of PARP and caspase 3 in the CD138^+^ and/or CD138^−^ populations. Each lane was loaded with 10 µg of protein. (**D**) A mechanistic model of circumvention of Mcl-1-dependent drug resistance by the Chk1/MEK inhibitor regimen. Mcl-1 plays an important role in both the survival of MM cell and sensitivity to various anti-MM agents, including bortezomib, as well as contributing to microenvironmental forms of drug resistance. A regimen combining a MEK1/2 inhibitor (MEKi) and a Chk1 inhibitor (Chk1i) acts at multiple levels in MM cells displaying Mcl-1-dependent bortezomib resistance, including a) down-regulation of Mcl-1 through a transcriptional mechanism; b) up-regulation of Bim and increased Bim/Mcl-1 binding, accompanied by release and activation of Bak and Bax; c) induction of MOMP (mitochondrial outer membrane permeabilization) and apoptosis; and d) possibly alternative Mcl-1-independent mechanism(s) of action (MOAs).

Lastly, the effects of this regimen were tested in primary MM samples. Co-exposure to CEP3891 and PD184352 resulted in significant increases in cell death in CD138^+^ MM cells isolated from 8 of 9 primary samples analyzed (**Fig. 6A**), but exerted minimal toxicity toward their CD138^−^ counterparts (**Fig. 6B**). Notably, in one sample (#13) in which a sufficient number of CD138^+^ cells were available for Western blot analysis, combined treatment induced marked Mcl-1 down-regulation, associated with caspase-3 and PARP cleavage in CD138^+^ cells (**Fig. 6C**). Interestingly, CD138^−^ cells exhibited minimal basal Mcl-1 level and little evidence of PARP or caspase-3 cleavage after drug treatment. Mcl-1 down-regulation following combined treatment was validated in two additional CD138^+^ samples (**Fig. 6C** lower and **Fig. S6E**). Moreover, the lack of toxicity of the regimen to non-neoplastic cells was also observed in normal human CD34^+^ cells (**Fig. S6F**). These findings raise the possibility that the MEK/Chk1 inhibitor regimen may act selectively against MM cells.

## Discussion

Mcl-1 has been implicated in the development of diverse malignancies, including those of hematopoietic origin such as MM, mantle cell lymphoma, and acute myelogenous leukemia [3], [50], [51]. In particular, it plays an important role in the survival of MM cells [4], [5], [10], as well as in the development of resistance to proteasome inhibitors such as bortezomib [7]–[9], a class of agents that are highly active as first-line treatment for patients with MM. Moreover, Mcl-1 up-regulation has been linked to microenvironmental stromal cell-related drug resistance in MM [17], [48]. Targeting Mcl-1, a short half-life protein [6], induces rapid apoptosis in MM cells, even with continuous expression of other anti-apoptotic proteins [3], [4]. Therefore, the central regulatory role of Mcl-1 as a survival and proliferation checkpoint factor makes this protein an attractive target for therapeutic intervention in MM [3], [5]. Because Mcl-1 abundance is reciprocally regulated by gene expression at multiple levels (e.g., transcriptional, translational) as well as proteasomal degradation [5], [6], various strategies have been employed to suppress its expression, including the use of transcriptional, translational, and deubiquitinase inhibitors [21], [23], [52]. In addition, phosphorylation influences interactions between Mcl-1 and pro-apoptotic proteins (e.g., Bim and Bak), thereby modifying its anti-apoptotic functions [53], [54]. The present results demonstrate for the first time that a strategy combining Chk1 with MEK1/2 inhibitors effectively kills MM cells, including those exhibiting Mcl-1 up-regulation and acquired resistance to bortezomib, as well as MM cells cultured in the presence of microenvironmental factors known to confer resistance to standard chemotherapeutic agents. They also raise the possibility that this combination regimen may act synergistically due to cooperative effects including down-regulation of Mcl-1 (by the Chk1 inhibitor CEP3891) and disabling of Mcl-1 anti-apoptotic functions (e.g., sequestration of Bak) in association with Bim up-regulation and increased Bim/Mcl-1 binding (by the MEK1/2 inhibitor PD184352). However, while the contribution of these events to the activity of this regimen in Mcl-1-overexpressing, bortezomib-resistant cells appears plausible, an alternative explanation e.g., that this regimen acts by triggering one or more Mcl-1-independent cell death pathways cannot presently be excluded. Efforts to investigate this possibility are currently underway.

The mechanism by which CEP3891 reduced expression of Mcl-1 remains to be fully elucidated, but appears to involve, at least in part, inhibition of gene transcription. On the other hand, interruption of the MEK1/2/ERK1/2 pathway by PD184352 is known to up-regulate Bim through a post-translational mechanism [40], [55]. Together, these actions may act in concert to attenuate Mcl-1 anti-apoptotic functions. The balance between Mcl-1 and Bim levels has been identified as a critical determinant of MM cell fate [56]. In this context, Bim up-regulation by MEK1/2 inhibition increased the amount of protein available for binding to Mcl-1, an event reported to disrupt Mcl-1 function [34]. Indeed, the current strategy increased the amount of Bim bound to Mcl-1, accompanied by release of Bak from Mcl-1 and activation of Bak and Bax. Consistent with these results, recent studies indicate that alterations in the associations/interactions between pro- and anti-apoptotic proteins may play a role in determining cell fate [32], [46]. Thus, the finding that combined treatment with CEP3891 and PD184352 was active against cells ectopically expressing Mcl-1, which confers striking bortezomib resistance, suggests that the MEK/Chk1 inhibitor strategy may effectively circumvent Mcl-1-dependent drug resistance.

Interpretation of the impact of Bim/Mcl-1 binding has differed in the literature, possibly reflecting cell type- and stimulus-dependent phenomena. For example, in Jurkat leukemia cells, disruption of Bim/Mcl-1 binding has been postulated to contribute to granzyme B-mediated apoptosis [57]. Moreover, bortezomib has been reported to induce apoptosis in myeloma cells by dissociation of Bim/Mcl-1 complexes, most likely through Noxa induction [47]. In contrast, an increase in the Bim/Mcl-1 association has been associated with enhanced apoptosis in leukemia cells co-exposed to BH3 mimetics and MEK1/2 inhibitors [35]. Moreover, rescue of fibroblasts from serum deprivation-induced cell death by growth factors has been attributed to Bim/Mcl-1 dissociation due to ERK1/2 activation [33]. In this context, the interaction between Bim and pro-survival Bcl-2 family proteins (e.g., Mcl-1) is regulated by ERK1/2-dependent phosphorylation of Bim [34]. This phenomenon has been attributed to promotion of Bim degradation following its release from Mcl-1, as well as preservation of Mcl-1 anti-apoptotic actions [33], [34]. The present findings are compatible with the latter mechanism in that an increased binding between Mcl-1 and Bim was associated with release of Bak from Mcl-1, even in cells over-expressing Mcl-1. Together, these results suggest that as in the case of MEK1/2 inhibition [33], [34], increased Bim/Mcl-1 association is likely to play a pro-apoptotic role in the ability of this regimen to circumvent Mcl-1-dependent drug resistance in MM cells.

The bone marrow microenvironment, composed of BMSCs, stromal factors (including cytokines and growth factors), and extracellular matrix proteins, is essential for the survival and growth of MM cells as well as resistance to diverse therapies [11], [15]. Although the precise role of Mcl-1 in stromal cell-mediated drug resistance has not yet been clearly defined, it is known that MM cells adhere to and induce bone marrow stromal cells (BMSCs) to secret multiple stromal factors (e.g., IL-6), which in turn promote MM cell survival [3], [5]. Mcl-1 is required for both VEGF and IL-6-promoted MM survival and proliferation [16]–[18]. Moreover, stromal factors (e.g., IL-6) have also been implicated in resistance of MM cells to both conventional cytotoxic drugs and novel targeted agents [11], [49], [58]. Furthermore, drug resistance conferred by various stromal factors (e.g., IL-6) has been related, at least in part, to Mcl-1 up-regulation in MM cells [18], [59]. Another major mechanism underlying the cytoprotective actions of stromal factors involves activation of the MEK1/2/ERK1/2 pathway, which leads to Bim phosphorylation and proteasomal degradation [16], [58], [60]. Consequently, MEK1/2 inhibitors have been reported to overcome bone marrow stromal factor-mediated drug resistance in MM cells [60], [61]. In this context, the MEK/Chk1 inhibitor strategy, which both down-regulates Mcl-1 and up-regulates Bim, may act in a cooperative manner to overcome BMSC- and stromal factor-mediated drug resistance. Indeed, this regimen was fully active against MM cells cultured in the presence of stromal factors (e.g., IL-6 and IGF-1), stromal cell-derived conditioned medium, or BMSCs. Notably, the regimen retained its ability to down-regulate Mcl-1 and up-regulate Bim in the presence of these microenvironmental factors that confer resistance to conventional anti-MM agents.

Multiple mechanisms of resistance to proteasome inhibitors such as bortezomib have been described, including mutation or amplification of proteasome sub-units, up-regulation of anti-oxidant proteins, and overexpression of anti-apoptotic proteins, etc. [2], [62], [63]. Among these mechanisms, up-regulation of Mcl-1 has often been implicated in proteasome inhibitor resistance [7], [9]. For example, administration of proteasome inhibitors (e.g., bortezomib) induce Mcl-1 accumulation by blocking its proteasomal degradation [64], thus limiting their anti-MM activity [7], [8]. Consistent with these findings, MM cells ectopically expressing Mcl-1 were highly resistant to bortezomib, while bortezomib-resistant MM cells (e.g., PS-R cells), which acquired resistance through continuous culture in progressively higher bortezomib concentrations, exhibited both Mcl-1 up-regulation and Bim down-regulation [20]. Significantly, neither of these cells displayed cross-resistance to MEK/Chk1 inhibition. Importantly, the MEK/Chk1inhibitor strategy was able to release Bak from Mcl-1 in both drug-naïve and bortezomib-resistant MM cells. Under normal conditions, Bak is held in check by its inhibitory associations with both Mcl-1 and Bcl-xL [65], while interventions that down-regulate Mcl-1 untether Bak, leading to Bak activation and apoptosis [24], [34]. Of note, Chk1/MEK1/2 inhibition also untethered Bak from Mcl-1, and triggered Bak activation in bortezomib-resistant MM cells either endogenously displaying high levels of or ectopically expressing Mcl-1.

It is noteworthy that the MEK/Chk1 inhibitor regimen also down-regulated Mcl-1 in primary CD138^+^ MM cells. Interestingly, basal Mcl-1 levels were not detectable in non-malignant bone marrow CD138^−^ cells. It is therefore tempting to speculate that higher basal expression of Mcl-1 in MM cells reflects the dependence of neoplastic cells on this protein for survival. This notion is supported by evidence that high Mcl-1 expression discriminates between primary MM versus normal cells, and also correlates with disease progression and clinical outcome [10]. If validated, this mechanism could potentially account for the preferential lethality of the regimen towards MM cells. However, additional studies will be required to establish the basis for this selectivity more definitively. Such studies are currently underway.

In summary, the present findings demonstrate that a strategy combining Chk1 with MEK1/2 inhibitors is shows pronounced activity against MM cells with acquired bortezomib-resistance or ectopically expressing high levels of Mcl-1, an anti-apoptotic protein which has been implicated in resistance to numerous anti-MM agents including bortezomib [8], [9] as well as in drug resistance conferred by microenvironmental factors [15], [48]. A hypothetical model outlining these mechanisms is summarized in (**Fig. 6D**). According to this model, up-regulation of Mcl-1 contributes to acquired bortezomib-resistance and the pro-survival effects of microenvironmental factors. The MEK/Chk1 inhibitor combination strategy acts through Mcl-1 down-regulation (e.g., by CEP3891), as well as Bim up-regulation (e.g., by PD184352), which increases the binding of Bim to Mcl-1 and unleashes Bak from Mcl-1. These events act cooperatively to trigger mitochondrial membrane permeabilization, leading to caspase activation and apoptosis. Finally, an additional possibility exists that the Chk1/MEK1/2 inhibitor regimen may trigger Mcl-1-independent cell death pathways. Collectively, these findings provide evidence arguing for an important role for Mcl-1 in multiple forms of drug resistance (e.g., acquired bortezomib- and microenvironmental factor-mediated drug resistance) in MM cells. They also raise the possibility that a strategy combining Chk1 with MEK1/2 inhibitors may be effective against various forms of Mcl-1-related drug-resistance. Accordingly, efforts to pursue this strategy further in humans are in development.

## Supporting Information

Figure S1 **The PD184352/CEP3891 regimen up-regulates Bim and induces apoptosis in a dose-dependent manner in various multiple myeloma cells.**
(TIF)

Figure S2 **CEP3891/PD184352 transcriptionally down-regulates Mcl-1, while ectopic over-expression of Mcl-1 fails to prevent cell death.**
(TIF)

Figure S3 **PD184352/CEP3891 down-regulates Mcl-1 in bortezomib-resistant myeloma cells.**
(TIF)

Figure S4 **The PD184352/CEP3891 regimen is active against bortezomib-resistant OPM-2 cells.**
(TIF)

Figure S5 **The PD184352/CEP3891 regimen overcomes BMSC-mediated drug-resistance.**
(TIF)

Figure S6 **The PD184352/CEP3891 regimen diminishes the colony-forming ability of myeloma cells in the presence or absence of stromal cells.**
(TIF)

## References

[1] Laubach JP, Mitsiades CS, Mahindra A, Luskin MR, Rosenblatt J, et al (2011) Management of relapsed and relapsed/refractory multiple myeloma. J Natl Compr Canc Netw 9: 1209–1216 9/10/1209 [pii]. 21975917 10.6004/jnccn.2011.0098

[2] Mahindra A, Laubach J, Raje N, Munshi N, Richardson PG, et al (2012) Latest advances and current challenges in the treatment of multiple myeloma. Nat Rev Clin Oncol 9: 135–143 nrclinonc.2012.15 [pii];10.1038/nrclinonc.2012.15 [doi] 22349016 10.1038/nrclinonc.2012.15

[3] Zhang B, Gojo I, Fenton RG (2002) Myeloid cell factor-1 is a critical survival factor for multiple myeloma. Blood 99: 1885–1893. 11877256 10.1182/blood.v99.6.1885

[4] Derenne S, Monia B, Dean NM, Taylor JK, Rapp MJ, et al (2002) Antisense strategy shows that Mcl-1 rather than Bcl-2 or Bcl-x(L) is an essential survival protein of human myeloma cells. Blood 100: 194–199. 12070027 10.1182/blood.v100.1.194

[5] Le GS, Podar K, Harousseau JL, Anderson KC (2004) Mcl-1 regulation and its role in multiple myeloma. Cell Cycle 3: 1259–1262 1196 [pii]. 15467463 10.4161/cc.3.10.1196

[6] Yang-Yen HF (2006) Mcl-1: a highly regulated cell death and survival controller. J Biomed Sci 13: 201–204 10.1007/s11373-005-9064-4 [doi] 16456709

[7] Hu J, Dang N, Menu E, De BE, Xu D, et al (2012) Activation of ATF4 mediates unwanted Mcl-1 accumulation by proteasome inhibition. Blood 119: 826–837 blood-2011-07-366492 [pii];10.1182/blood-2011-07-366492 [doi] 22128141 10.1182/blood-2011-07-366492

[8] Nencioni A, Hua F, Dillon CP, Yokoo R, Scheiermann C, et al (2005) Evidence for a protective role of Mcl-1 in proteasome inhibitor-induced apoptosis. Blood 105: 3255–3262 2004-10-3984 [pii];10.1182/blood-2004-10-3984 [doi] 15613543 10.1182/blood-2004-10-3984

[9] Gomez-Bougie P, Wuilleme-Toumi S, Menoret E, Trichet V, Robillard N, et al (2007) Noxa up-regulation and Mcl-1 cleavage are associated to apoptosis induction by bortezomib in multiple myeloma. Cancer Res 67: 5418–5424 67/11/5418 [pii];10.1158/0008-5472.CAN-06-4322 [doi]. 17545623 10.1158/0008-5472.CAN-06-4322

[10] Wuilleme-Toumi S, Robillard N, Gomez P, Moreau P, Le GS, et al (2005) Mcl-1 is overexpressed in multiple myeloma and associated with relapse and shorter survival. Leukemia 19: 1248–1252 2403784 [pii];10.1038/sj.leu.2403784 [doi] 15902294 10.1038/sj.leu.2403784

[11] McMillin DW, Delmore J, Weisberg E, Negri JM, Geer DC, et al (2010) Tumor cell-specific bioluminescence platform to identify stroma-induced changes to anticancer drug activity. Nat Med 16: 483–489 nm.2112 [pii];10.1038/nm.2112 [doi] 20228816 10.1038/nm.2112 PMC3786785

[12] Pagnucco G, Cardinale G, Gervasi F (2004) Targeting multiple myeloma cells and their bone marrow microenvironment. Ann N Y Acad Sci 1028: 390–399 1028/1/390 [pii];10.1196/annals.1322.047 [doi] 15650264 10.1196/annals.1322.047

[13] Hazlehurst LA, Landowski TH, Dalton WS (2003) Role of the tumor microenvironment in mediating de novo resistance to drugs and physiological mediators of cell death. Oncogene 22: 7396–7402 10.1038/sj.onc.1206943 [doi];1206943 [pii] 14576847

[14] Nair RR, Gebhard AW, Emmons MF, Hazlehurst LA (2012) Emerging strategies for targeting cell adhesion in multiple myeloma. Adv Pharmacol 65: 143–189 B978-0-12-397927-8.00006-3 [pii];10.1016/B978-0-12-397927-8.00006-3 [doi] 22959026 10.1016/B978-0-12-397927-8.00006-3

[15] Mitsiades CS, Mitsiades NS, Munshi NC, Richardson PG, Anderson KC (2006) The role of the bone microenvironment in the pathophysiology and therapeutic management of multiple myeloma: interplay of growth factors, their receptors and stromal interactions. Eur J Cancer 42: 1564–1573 S0959-8049(06)00317-0 [pii];10.1016/j.ejca.2005.12.025 [doi] 16765041 10.1016/j.ejca.2005.12.025

[16] Yasui H, Hideshima T, Richardson PG, Anderson KC (2006) Novel therapeutic strategies targeting growth factor signalling cascades in multiple myeloma. Br J Haematol 132: 385–397 BJH5860 [pii];10.1111/j.1365-2141.2005.05860.x [doi] 16412014 10.1111/j.1365-2141.2005.05860.x

[17] Le GS, Podar K, Amiot M, Hideshima T, Chauhan D, et al (2004) VEGF induces Mcl-1 up-regulation and protects multiple myeloma cells against apoptosis. Blood 104: 2886–2892 10.1182/blood-2004-05-1760 [doi];2004-05-1760 [pii] 15217829

[18] Jourdan M, Veyrune JL, De VJ, Redal N, Couderc G, et al (2003) A major role for Mcl-1 antiapoptotic protein in the IL-6-induced survival of human myeloma cells. Oncogene 22: 2950–2959 10.1038/sj.onc.1206423 [doi];1206423 [pii] 12771946 PMC2396235

[19] Nguyen M, Marcellus RC, Roulston A, Watson M, Serfass L, et al (2007) Small molecule obatoclax (GX15-070) antagonizes MCL-1 and overcomes MCL-1-mediated resistance to apoptosis. Proc Natl Acad Sci U S A 104: 19512–19517 0709443104 [pii];10.1073/pnas.0709443104 [doi] 18040043 10.1073/pnas.0709443104 PMC2148320

[20] Chen S, Dai Y, Pei XY, Myers J, Wang L, et al (2012) CDK inhibitors upregulate BH3-only proteins to sensitize human myeloma cells to BH3 mimetic therapies. Cancer Res 72: 4225–4237 0008-5472.CAN-12-1118 [pii];10.1158/0008-5472.CAN-12-1118 [doi] 22693249 10.1158/0008-5472.CAN-12-1118 PMC3421040

[21] Fulda S (2012) Shifting the balance of mitochondrial apoptosis: therapeutic perspectives. Front Oncol 2: 121 10.3389/fonc.2012.00121 [doi] 23061040 PMC3465793

[22] MacCallum DE, Melville J, Frame S, Watt K, Anderson S, et al (2005) Seliciclib (CYC202, R-Roscovitine) induces cell death in multiple myeloma cells by inhibition of RNA polymerase II-dependent transcription and down-regulation of Mcl-1. Cancer Res 65: 5399–5407 65/12/5399 [pii];10.1158/0008-5472.CAN-05-0233 [doi] 15958589 10.1158/0008-5472.CAN-05-0233

[23] Rahmani M, Davis EM, Bauer C, Dent P, Grant S (2005) Apoptosis induced by the kinase inhibitor BAY 43-9006 in human leukemia cells involves down-regulation of Mcl-1 through inhibition of translation. J Biol Chem 280: 35217–35227 M506551200 [pii];10.1074/jbc.M506551200 [doi] 16109713 10.1074/jbc.M506551200

[24] Chen S, Dai Y, Harada H, Dent P, Grant S (2007) Mcl-1 down-regulation potentiates ABT-737 lethality by cooperatively inducing Bak activation and Bax translocation. Cancer Res 67: 782–791 67/2/782 [pii];10.1158/0008-5472.CAN-06-3964 [doi] 17234790 10.1158/0008-5472.CAN-06-3964

[25] Bhat UG, Pandit B, Gartel AL (2010) ARC synergizes with ABT-737 to induce apoptosis in human cancer cells. Mol Cancer Ther 9: 1688–1696 1535-7163.MCT-09-0919 [pii];10.1158/1535-7163.MCT-09-0919 [doi] 20515947 10.1158/1535-7163.MCT-09-0919 PMC2902270

[26] Perez-Galan P, Roue G, Villamor N, Campo E, Colomer D (2007) The BH3-mimetic GX15-070 synergizes with bortezomib in mantle cell lymphoma by enhancing Noxa-mediated activation of Bak. Blood 109: 4441–4449 blood-2006-07-034173 [pii];10.1182/blood-2006-07-034173 [doi] 17227835 10.1182/blood-2006-07-034173

[27] Eichhorn JM, Alford SE, Hughes CC, Fenical W, Chambers TC (2013) Purported Mcl-1 inhibitor marinopyrrole A fails to show selective cytotoxicity for Mcl-1-dependent cell lines. Cell Death Dis 4: e880 cddis2013411 [pii];10.1038/cddis.2013.411 [doi] 24157874 10.1038/cddis.2013.411 PMC3920948

[28] Dai Y, Chen S, Pei XY, Almenara JA, Kramer LB, et al (2008) Interruption of the Ras/MEK/ERK signaling cascade enhances Chk1 inhibitor-induced DNA damage in vitro and in vivo in human multiple myeloma cells. Blood 112: 2439–2449. 18614762 10.1182/blood-2008-05-159392 PMC2532812

[29] Syljuasen RG, Sorensen CS, Hansen LT, Fugger K, Lundin C, et al (2005) Inhibition of human Chk1 causes increased initiation of DNA replication, phosphorylation of ATR targets, and DNA breakage. Mol Cell Biol 25: 3553–3562. 15831461 10.1128/MCB.25.9.3553-3562.2005 PMC1084285

[30] Dai Y, Landowski TH, Rosen ST, Dent P, Grant S (2002) Combined treatment with the checkpoint abrogator UCN-01 and MEK1/2 inhibitors potently induces apoptosis in drug-sensitive and -resistant myeloma cells through an IL-6-independent mechanism. Blood 100: 3333–3343. 12384435 10.1182/blood-2002-03-0940

[31] Dai Y, Chen S, Shah R, Pei XY, Wang L, et al (2011) Disruption of Src function potentiates Chk1-inhibitor-induced apoptosis in human multiple myeloma cells in vitro and in vivo. Blood 117: 1947–1957 blood-2010-06-291146 [pii];10.1182/blood-2010-06-291146 [doi] 21148814 10.1182/blood-2010-06-291146 PMC3056642

[32] Kawabata T, Tanimura S, Asai K, Kawasaki R, Matsumaru Y, et al (2012) Up-regulation of pro-apoptotic protein Bim and down-regulation of anti-apoptotic protein Mcl-1 cooperatively mediate enhanced tumor cell death induced by the combination of ERK kinase (MEK) inhibitor and microtubule inhibitor. J Biol Chem 287: 10289–10300 M111.319426 [pii];10.1074/jbc.M111.319426 [doi] 22270368 10.1074/jbc.M111.319426 PMC3322996

[33] Ewings KE, Hadfield-Moorhouse K, Wiggins CM, Wickenden JA, Balmanno K, et al (2007) ERK1/2-dependent phosphorylation of BimEL promotes its rapid dissociation from Mcl-1 and Bcl-xL. EMBO J 26: 2856–2867. 17525735 10.1038/sj.emboj.7601723 PMC1894764

[34] Ewings KE, Wiggins CM, Cook SJ (2007) Bim and the pro-survival Bcl-2 proteins: opposites attract, ERK repels. Cell Cycle 6: 2236–2240 4728 [pii]. 17881896 10.4161/cc.6.18.4728

[35] Konopleva M, Milella M, Ruvolo P, Watts JC, Ricciardi MR, et al (2012) MEK inhibition enhances ABT-737-induced leukemia cell apoptosis via prevention of ERK-activated MCL-1 induction and modulation of MCL-1/BIM complex. Leukemia 26: 778–787 leu2011287 [pii];10.1038/leu.2011.287 [doi] 22064351 10.1038/leu.2011.287 PMC3604791

[36] Pei XY, Dai Y, Youssefian LE, Chen S, Bodie WW, et al (2011) Cytokinetically quiescent (G0/G1) human multiple myeloma cells are susceptible to simultaneous inhibition of Chk1 and MEK1/2. Blood 118: 5189–5200 blood-2011-02-339432 [pii];10.1182/blood-2011-02-339432 [doi] 21911831 10.1182/blood-2011-02-339432 PMC3217403

[37] Zhang J, Choi Y, Mavromatis B, Lichtenstein A, Li W (2003) Preferential killing of PTEN-null myelomas by PI3K inhibitors through Akt pathway. Oncogene 22: 6289–6295 10.1038/sj.onc.1206718 [doi];1206718 [pii] 13679867

[38] Moalli PA, Pillay S, Krett NL, Rosen ST (1993) Alternatively spliced glucocorticoid receptor messenger RNAs in glucocorticoid-resistant human multiple myeloma cells. Cancer Res 53: 3877–3879. 8358712

[39] Dai Y, Chen S, Wang L, Pei XY, Funk VL, et al (2011) Disruption of IkappaB kinase (IKK)-mediated RelA serine 536 phosphorylation sensitizes human multiple myeloma cells to histone deacetylase (HDAC) inhibitors. J Biol Chem 286: 34036–34050 M111.284216 [pii];10.1074/jbc.M111.284216 [doi] 21816815 10.1074/jbc.M111.284216 PMC3190767

[40] Pei XY, Dai Y, Tenorio S, Lu J, Harada H, et al (2007) MEK1/2 inhibitors potentiate UCN-01 lethality in human multiple myeloma cells through a Bim-dependent mechanism. Blood 110: 2092–2101. 17540843 10.1182/blood-2007-04-083204 PMC1976370

[41] Schmidmaier R, Baumann P, Meinhardt G (2006) Cell-cell contact mediated signalling - no fear of contact. Exp Oncol 28: 12–15 25/488 [pii]. 16614701

[42] Schmidmaier R, Baumann P, Simsek M, Dayyani F, Emmerich B, et al (2004) The HMG-CoA reductase inhibitor simvastatin overcomes cell adhesion-mediated drug resistance in multiple myeloma by geranylgeranylation of Rho protein and activation of Rho kinase. Blood 104: 1825–1832 10.1182/blood-2003-12-4218 [doi];2003-12-4218 [pii] 15161667

[43] Hsu YT, Youle RJ (1997) Nonionic detergents induce dimerization among members of the Bcl-2 family. J Biol Chem 272: 13829–13834. 9153240 10.1074/jbc.272.21.13829

[44] Chou TC, Talalay P (1984) Quantitative analysis of dose-effect relationships: the combined effects of multiple drugs or enzyme inhibitors. Adv Enzyme Regul 22: 27–55. 6382953 10.1016/0065-2571(84)90007-4

[45] Senderowicz AM (2000) Small molecule modulators of cyclin-dependent kinases for cancer therapy. Oncogene 19: 6600–6606 10.1038/sj.onc.1204085 [doi] 11426645

[46] Kroemer G, Galluzzi L, Brenner C (2007) Mitochondrial membrane permeabilization in cell death. Physiol Rev 87: 99–163 87/1/99 [pii];10.1152/physrev.00013.2006 [doi] 17237344 10.1152/physrev.00013.2006

[47] Gomez-Bougie P, Wuilleme-Toumi S, Menoret E, Trichet V, Robillard N, et al (2007) Noxa up-regulation and Mcl-1 cleavage are associated to apoptosis induction by bortezomib in multiple myeloma. Cancer Res 67: 5418–5424 67/11/5418 [pii];10.1158/0008-5472.CAN-06-4322 [doi] 17545623 10.1158/0008-5472.CAN-06-4322

[48] Manier S, Sacco A, Leleu X, Ghobrial IM, Roccaro AM (2012) Bone marrow microenvironment in multiple myeloma progression. J Biomed Biotechnol 2012: 157496 10.1155/2012/157496 [doi] 23093834 PMC3471001

[49] Nefedova Y, Landowski TH, Dalton WS (2003) Bone marrow stromal-derived soluble factors and direct cell contact contribute to de novo drug resistance of myeloma cells by distinct mechanisms. Leukemia 17: 1175–1182 10.1038/sj.leu.2402924 [doi];2402924 [pii] 12764386

[50] Khoury JD, Medeiros LJ, Rassidakis GZ, McDonnell TJ, Abruzzo LV, et al (2003) Expression of Mcl-1 in mantle cell lymphoma is associated with high-grade morphology, a high proliferative state, and p53 overexpression. J Pathol 199: 90–97 10.1002/path.1254 [doi] 12474231 10.1002/path.1254

[51] Gores GJ, Kaufmann SH (2012) Selectively targeting Mcl-1 for the treatment of acute myelogenous leukemia and solid tumors. Genes Dev 26: 305–311 26/4/305 [pii];10.1101/gad.186189.111 [doi] 22345513 10.1101/gad.186189.111 PMC3289878

[52] Bhat UG, Gartel AL (2010) Nucleoside analog ARC targets Mcl-1 to induce apoptosis in leukemia cells. Leukemia 24: 851–855 leu20103 [pii];10.1038/leu.2010.3 [doi] 20164850 10.1038/leu.2010.3 PMC3201742

[53] Nifoussi SK, Vrana JA, Domina AM, De BA, Gui J, et al (2012) Thr 163 phosphorylation causes Mcl-1 stabilization when degradation is independent of the adjacent GSK3-targeted phosphodegron, promoting drug resistance in cancer. PLoS One 7: e47060 10.1371/journal.pone.0047060 [doi];PONE-D-12-19117 [pii] 23056582 10.1371/journal.pone.0047060 PMC3467206

[54] Mazumder S, Choudhary GS, Al-Harbi S, Almasan A (2012) Mcl-1 Phosphorylation defines ABT-737 resistance that can be overcome by increased NOXA expression in leukemic B cells. Cancer Res 72: 3069–3079 0008-5472.CAN-11-4106 [pii];10.1158/0008-5472.CAN-11-4106 [doi] 22525702 10.1158/0008-5472.CAN-11-4106 PMC3377792

[55] Ley R, Ewings KE, Hadfield K, Howes E, Balmanno K, et al (2004) Extracellular signal-regulated kinases 1/2 are serum-stimulated “Bim(EL) kinases” that bind to the BH3-only protein Bim(EL) causing its phosphorylation and turnover. J Biol Chem 279: 8837–8847 10.1074/jbc.M311578200 [doi];M311578200 [pii] 14681225

[56] Morales AA, Kurtoglu M, Matulis SM, Liu J, Siefker D, et al (2011) Distribution of Bim determines Mcl-1 dependence or codependence with Bcl-xL/Bcl-2 in Mcl-1-expressing myeloma cells. Blood 118: 1329–1339 blood-2011-01-327197 [pii];10.1182/blood-2011-01-327197 [doi] 21659544 10.1182/blood-2011-01-327197 PMC3152498

[57] Han J, Goldstein LA, Gastman BR, Froelich CJ, Yin XM, et al (2004) Degradation of Mcl-1 by granzyme B: implications for Bim-mediated mitochondrial apoptotic events. J Biol Chem 279: 22020–22029 10.1074/jbc.M313234200 [doi];M313234200 [pii] 15014070

[58] Hideshima T, Mitsiades C, Tonon G, Richardson PG, Anderson KC (2007) Understanding multiple myeloma pathogenesis in the bone marrow to identify new therapeutic targets. Nat Rev Cancer 7: 585–598 nrc2189 [pii];10.1038/nrc2189 [doi] 17646864 10.1038/nrc2189

[59] Zhang B, Potyagaylo V, Fenton RG (2003) IL-6-independent expression of Mcl-1 in human multiple myeloma. Oncogene 22: 1848–1859 10.1038/sj.onc.1206358 [doi];1206358 [pii] 12660820

[60] Tai YT, Fulciniti M, Hideshima T, Song W, Leiba M, et al (2007) Targeting MEK induces myeloma-cell cytotoxicity and inhibits osteoclastogenesis. Blood 110: 1656–1663. 17510321 10.1182/blood-2007-03-081240 PMC1975848

[61] Kim K, Kong SY, Fulciniti M, Li X, Song W, et al (2010) Blockade of the MEK/ERK signalling cascade by AS703026, a novel selective MEK1/2 inhibitor, induces pleiotropic anti-myeloma activity in vitro and in vivo. Br J Haematol 149: 537–549 BJH8127 [pii];10.1111/j.1365-2141.2010.08127.x [doi] 20331454 10.1111/j.1365-2141.2010.08127.x PMC3418597

[62] Kuhn DJ, Berkova Z, Jones RJ, Woessner R, Bjorklund CC, et al (2012) Targeting the insulin-like growth factor-1 receptor to overcome bortezomib resistance in preclinical models of multiple myeloma. Blood 120: 3260–3270 blood-2011-10-386789 [pii];10.1182/blood-2011-10-386789 [doi] 22932796 10.1182/blood-2011-10-386789 PMC3476538

[63] Kale AJ, Moore BS (2012) Molecular mechanisms of acquired proteasome inhibitor resistance. J Med Chem 55: 10317–10327 10.1021/jm300434z [doi] 22978849 PMC3521846

[64] Bhat UG, Halasi M, Gartel AL (2009) FoxM1 is a general target for proteasome inhibitors. PLoS One 4: e6593 10.1371/journal.pone.0006593 [doi] 19672316 PMC2721658

[65] Willis SN, Chen L, Dewson G, Wei A, Naik E, et al (2005) Proapoptotic Bak is sequestered by Mcl-1 and Bcl-xL, but not Bcl-2, until displaced by BH3-only proteins. Genes Dev 19: 1294–1305 gad.1304105 [pii];10.1101/gad.1304105 [doi] 15901672 10.1101/gad.1304105 PMC1142553

