# Supplementary figures and images for: Circumvention of Mcl-1-Dependent Drug Resistance by Simultaneous Chk1 and MEK1/2 Inhibition in Human Multiple Myeloma Cells
Source: PLoS One. 2014 Mar 4;9(3):e89064. doi: 10.1371/journal.pone.0089064 (PMC3942309; doi:10.1371/journal.pone.0089064)

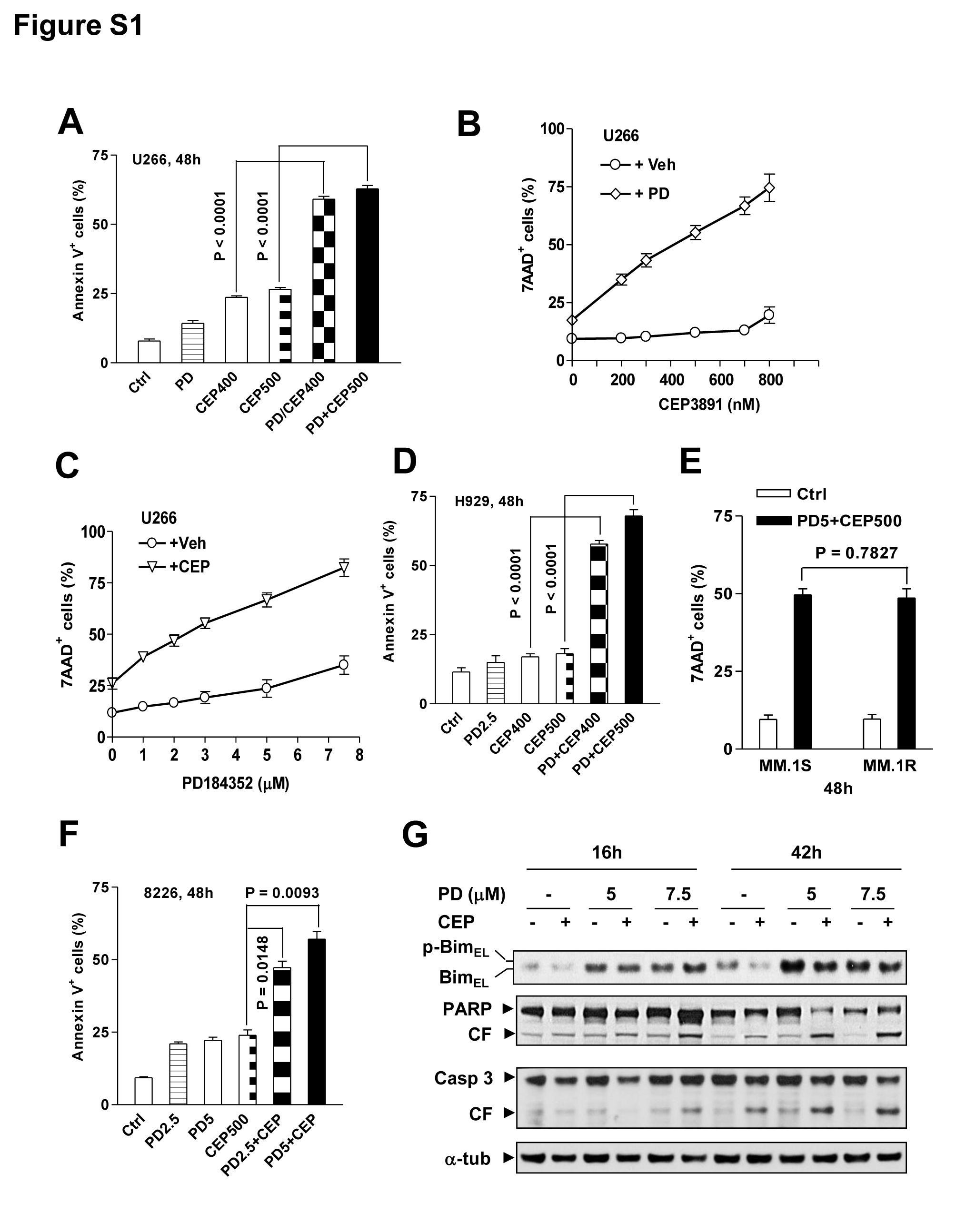

Supplement: Figure S1 — The PD184352/CEP3891 regimen up-regulates Bim and induces apoptosis in a dose-dependent manner in various multiple myeloma cells. (TIF) [file pone.0089064.s001.tif]

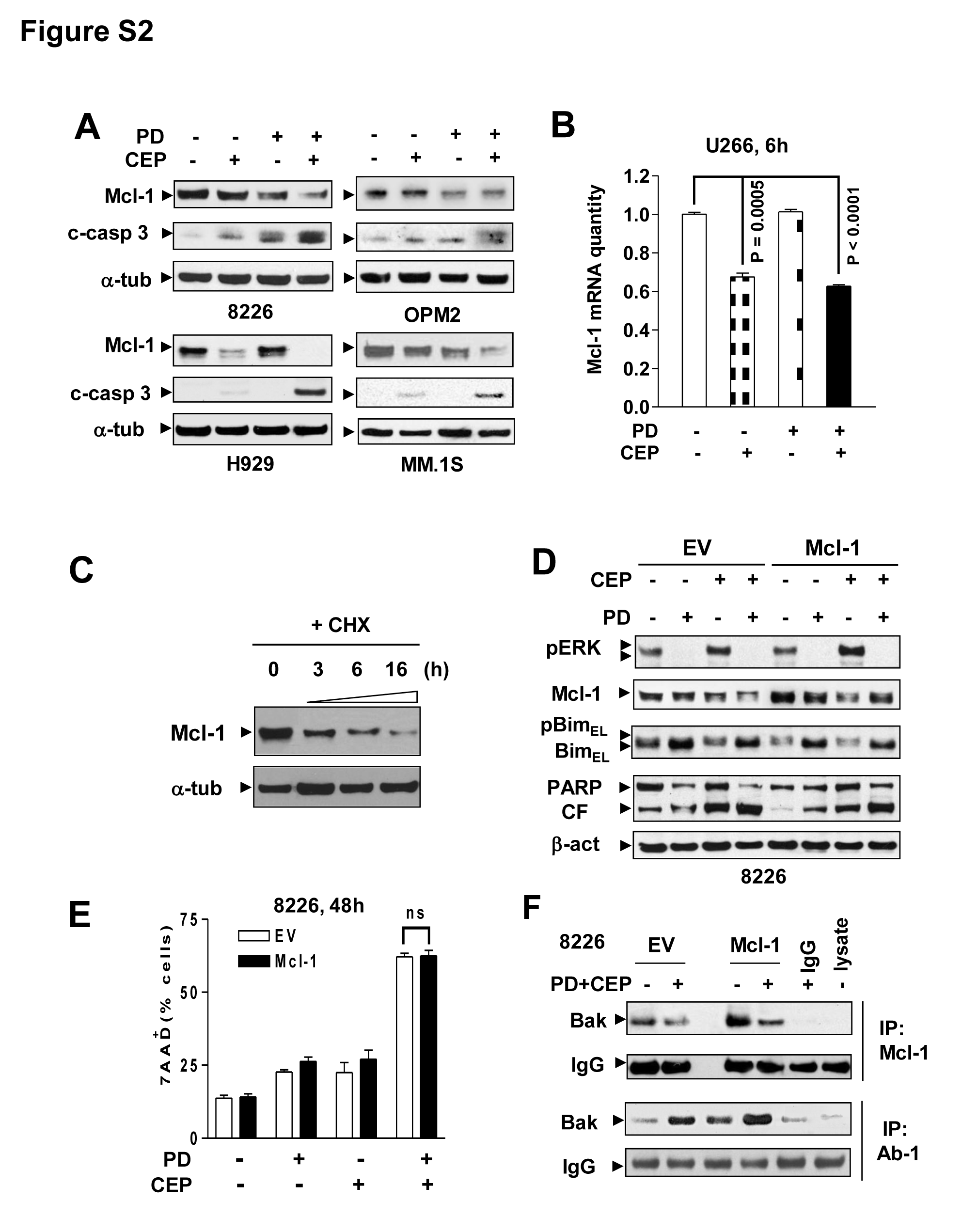

Supplement: Figure S2 — CEP3891/PD184352 transcriptionally down-regulates Mcl-1, while ectopic over-expression of Mcl-1 fails to prevent cell death. (TIF) [file pone.0089064.s002.tif]

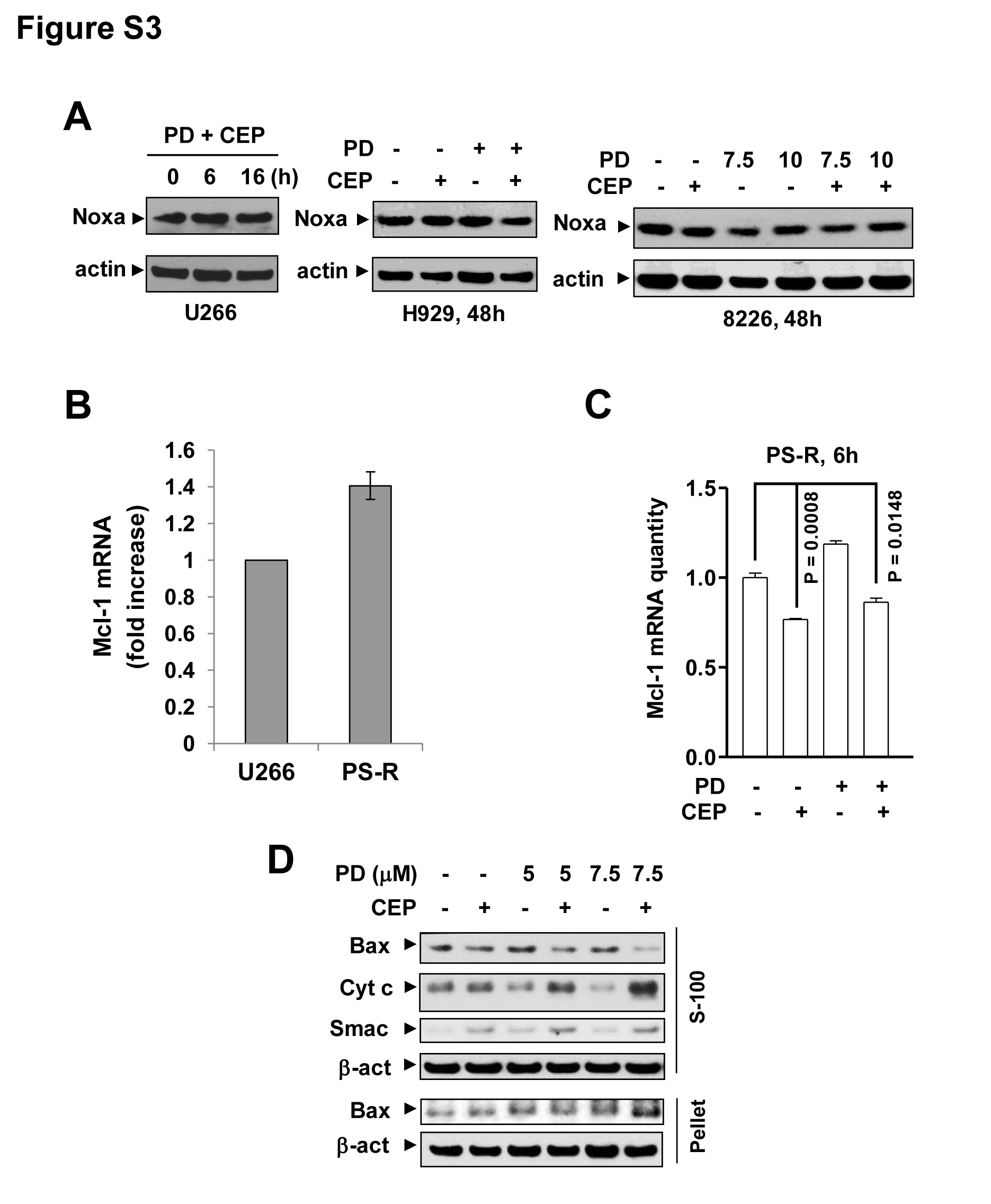

Supplement: Figure S3 — PD184352/CEP3891 down-regulates Mcl-1 in bortezomib-resistant myeloma cells. (TIF) [file pone.0089064.s003.tif]

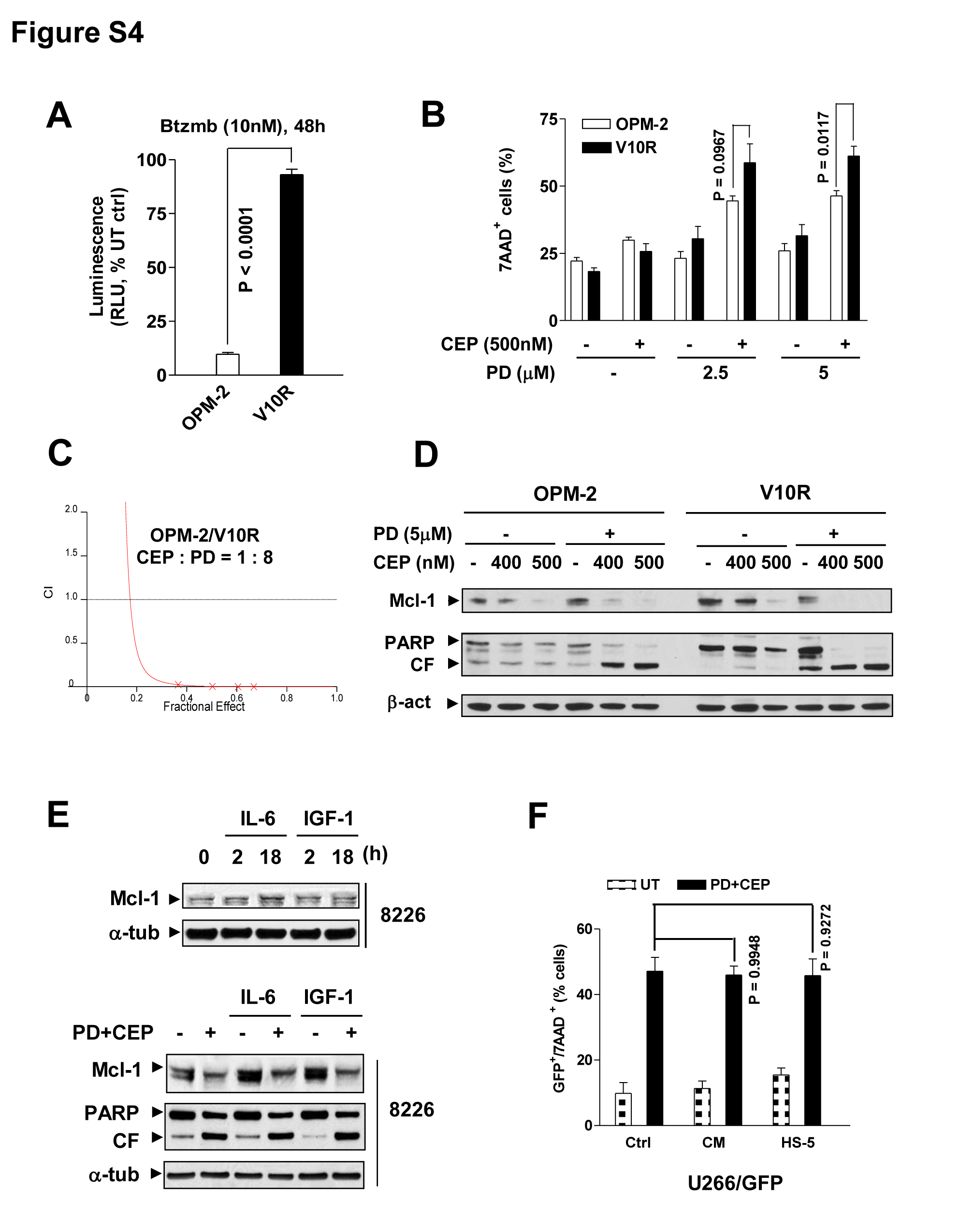

Supplement: Figure S4 — The PD184352/CEP3891 regimen is active against bortezomib-resistant OPM-2 cells. (TIF) [file pone.0089064.s004.tif]

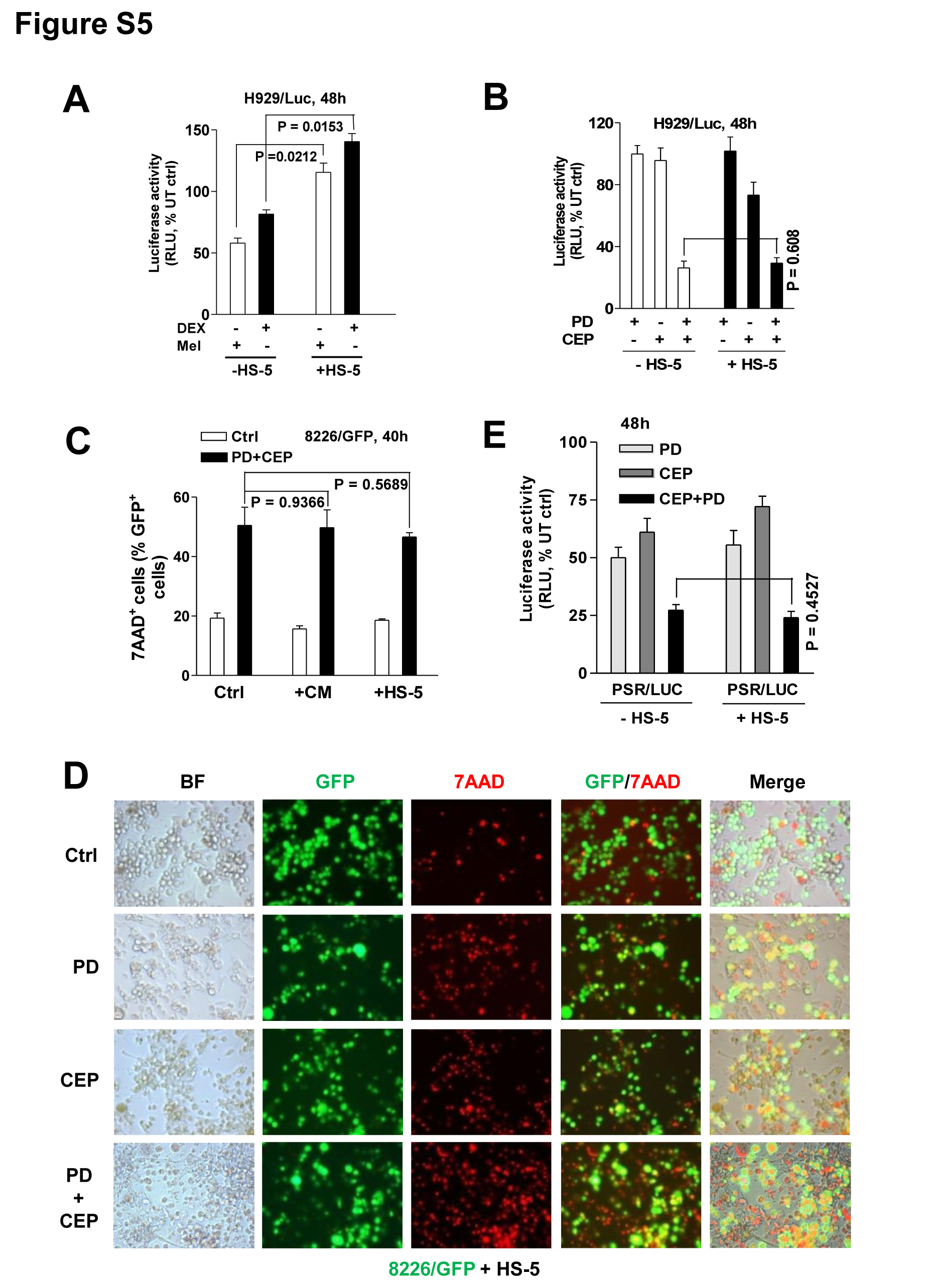

Supplement: Figure S5 — The PD184352/CEP3891 regimen overcomes BMSC-mediated drug-resistance. (TIF) [file pone.0089064.s005.tif]

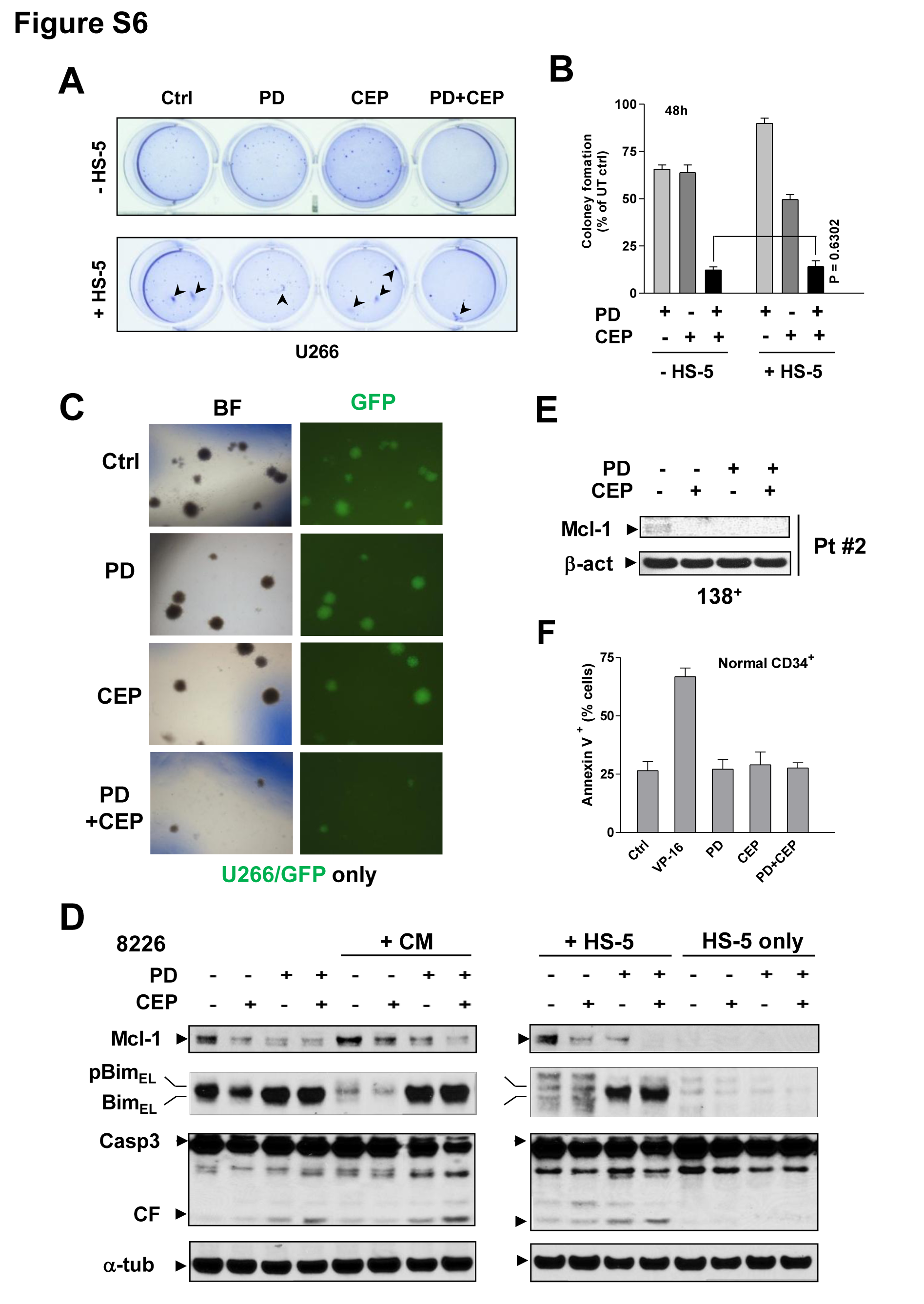

Supplement: Figure S6 — The PD184352/CEP3891 regimen diminishes the colony-forming ability of myeloma cells in the presence or absence of stromal cells. (TIF) [file pone.0089064.s006.tif]
